# Liquid–liquid phase separation of H3K27me3 reader BP1 regulates transcriptional repression

**DOI:** 10.1186/s13059-024-03209-7

**Published:** 2024-03-11

**Authors:** Guangfei Tang, Haoxue Xia, Yufei Huang, Yuanwen Guo, Yun Chen, Zhonghua Ma, Wende Liu

**Affiliations:** 1 State Key Laboratory for Biology of Plant Diseases and Insect Pests, Institute of Plant Protection, Chinese Academy of Agricultural Sciences, Beijing, 100193 China; 2 College of Plant Protection, Shenyang Agricultural University, Shenyang, 110866 China; 3 State Key Laboratory of Rice Biology, Key Laboratory of Molecular Biology of Crop Pathogens and Insects, Institute of Biotechnology, Zhejiang University, Hangzhou, 310058 China

## Abstract

**Background:**

Bromo-adjacent homology-plant homeodomain domain containing protein 1 (BP1) is a reader of histone post-translational modifications in fungi. BP1 recognizes trimethylation of lysine 27 in histone H3 (H3K27me3), an epigenetic hallmark of gene silencing. However, whether and how BP1 participates in transcriptional repression remains poorly understood.

**Results:**

We report that BP1 forms phase-separated liquid condensates to modulate its biological function in *Fusarium graminearum*. Deletion assays reveal that intrinsically disordered region 2 (IDR2) of BP1 mediates its liquid–liquid phase separation. The phase separation of BP1 is indispensable for its interaction with suppressor of Zeste 12, a component of polycomb repressive complex 2. Furthermore, IDR2 deletion abolishes BP1-H3K27me3 binding and alleviates the transcriptional repression of secondary metabolism-related genes, especially deoxynivalenol mycotoxin biosynthesis genes.

**Conclusions:**

BP1 maintains transcriptional repression by forming liquid–liquid phase-separated condensates, expanding our understanding of the relationship between post-translational modifications and liquid–liquid phase separation.

**Supplementary Information:**

The online version contains supplementary material available at 10.1186/s13059-024-03209-7.

## Background

Histone post-translational modifications (PTMs) are typically enriched at distinct genomic locations, where their presence alters chromatin structure or recruits downstream effectors, leading to changes in transcriptional activity at the molecular level [1]. Histone methylation is generally considered a highly stable and conserved PTM, occurring in both prokaryotes and eukaryotes [2]. Dynamic and reversible histone methylation is mediated by methyltransferases (so-called writers) and demethylases (erasers), responsible for catalyzing or removing these modifications, respectively, thus altering chromatin structure and regulating transcription [3]. Distinct histone methylation modifications are specifically recognized by histone-binding proteins called readers based on the methylation state and the surrounding amino acid sequence of histones [4]. It has been well documented that the effects of PTMs on gene expression can be mediated by such reader proteins [5].

Polycomb repressive complex 2 (PRC2) is a chromatin-associated methyltransferase complex that maintains transcriptional repression in eukaryotes by catalyzing lysine 27 trimethylation of histone H3 (H3K27me3), an epigenetic hallmark of gene silencing [6]. The PRC2 complex was initially identified in *Drosophila* (*Drosophila melanogaster*), including three core components: the histone methyltransferase enhancer of zeste 2 (Ezh2), embryonic ectoderm development (Eed), and suppressor of Zeste 12 (Suz12) [7]. Homologs of PRC2 subunits have been identified in different organisms, and the importance of PRC2 activity in regulating the expression of key development genes has been demonstrated [8]. Although the general repressive role of PRC2 has been retained during evolution, its functionality has substantially diversified [9]. The core PRC2 complex in the cereal fungal pathogen *Fusarium graminearum*, causing Fusarium head blight, was identified as a transcriptional repressor harboring these three core components based on homology to the *Drosophila* PRC2 proteins Kmt6 (H3K27me3 methyltransferase), Suz12, and Eed [10, 11]. Our recent work showed that bromo-adjacent homology (BAH)–plant homeodomain (PHD) domain-containing protein 1 (BP1) directly interacts with Suz12 and is a histone reader that recognizes H3K27me3 to maintain transcriptional repression in *F. graminearum* [10]. Although BP1 is recognized as a key reader for the H3K27me3 mark, how it recognizes H3K27me3 to regulate transcriptional repression remains unclear.

Liquid–liquid phase separation (LLPS) is a ubiquitous physicochemical phenomenon that underlies the formation of membrane-less intracellular compartments, regulating the spatiotemporal organization of proteins and nucleic acids in living cells [12]. Mounting evidence demonstrates that protein LLPS enables the highly efficient and reversible adjustment of cellular events such as chromatin organization, transcriptional regulation, and signal transduction [13]. Proteins that undergo LLPS often contain intrinsically disordered regions (IDRs) and remain dynamic in solution [14]. LLPS is driven by weak, multivalent interactions between macromolecules and can be regulated by effector molecules or PTMs [15]. *Drosophila* heterochromatin protein 1α (HP1α) displays LLPS properties that are critical for chromatin domain formation and overall genome function [16]. Human 53BP1 is a chromatin-binding protein that regulates DNA double-strand break repair and the maintenance of heterochromatin integrity through LLPS [17]. The plant-specific histone methyltransferase SUVR2 is shown to undergo LLPS to promote DNA repair in barrel clover (*Medicago truncatula*) [18]. In mammals, polycomb protein chromobox 2 (CBX2) forms liquid-like condensates that can concentrate DNA and nucleosomes [19]. Many plant and animal proteins form condensates in an LLPS-dependent manner to ensure optimal physiological activities. These proteins include Yin Yang 1 (YY1), methyl-CpG-binding protein 2 (MeCP2), SEUSS, zinc finger MYND-type containing 8 (ZMYND8), and ubiquitously transcribed X chromosome tetratricopeptide repeat protein (UTX) [20–24]. However, the biological function and precise regulatory mechanisms of LLPS in fungi remain poorly understood.

In this study, using a variety of biochemical and cell biology approaches, we show that the H3K27me3 reader BP1 contains two IDRs and dynamically forms nuclear puncta to undergo LLPS in *F. graminearum*. Notably, we demonstrate that IDR2 of BP1 mediates droplet formation, indicating that IDR2 promotes liquid-like properties and condensate formation in a biological context. Furthermore, we reveal that phase separation of BP1 regulates BP1–PRC2 interaction and H3K27me3 recognition to maintain transcriptional repression. Collectively, our results provide deeper knowledge of how PTM reader proteins specifically and efficiently regulate transcriptional repression through LLPS in fungi.

## Results

### BP1 represses DON biosynthesis-related gene expression

**Fig. 1:**
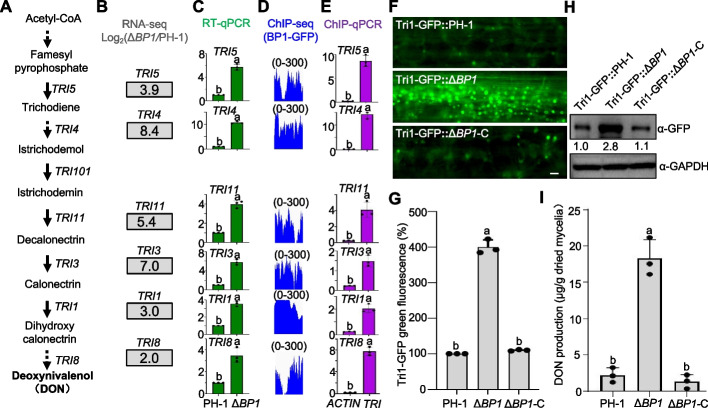
Loss of BP1 function upregulates DON production in *Fusarium graminearum*. **A** Diagram of the deoxynivalenol (DON) biosynthesis pathway. **B** Expression levels of *TRI* genes, as determined by transcriptome deep sequencing (RNA-seq; data were normalized to wild-type PH-1; Δ*BP1*/PH-1). **C** Relative transcript levels of *TRI* genes in wild-type PH-1 and Δ*BP1* as determined by reverse transcription quantitative PCR (RT-qPCR). Transcript levels were normalized to *ACTIN*, with levels in PH-1 set to 1. Different lowercase letters denote significant differences at *P* = 0.05. **D** Genome browser view of normalized BP1-GFP chromatin immunoprecipitation sequencing (ChIP-seq) peaks at representative *TRI* loci. The track scale is 0–300. **E** Verification of ChIP-seq results by ChIP-qPCR of the indicated *TRI* genes in Δ*BP1*::*BP1-GFP* (the complementation strain, Δ*BP1*-C) using an anti-GFP antibody. Different lowercase letters denote significant differences at *P* = 0.05. **F** Toxisome formation in the wild-type PH-1, Δ*BP1*, and Δ*BP1*-C strains inoculated on a wheat (*Triticum aestivum*) leaf for 2 days. **G** GFP signal intensity for each strain, with levels in PH-1 set to 1. Different lowercase letters denote significant differences at *P* = 0.05. **H** Immunoblot analysis of proteins isolated from the same set of samples used in **F**, detected with an anti-GFP antibody. GAPDH was used as a loading control. **I** DON contents in the wild-type PH-1, Δ*BP1*, and Δ*BP1*-C complement strains after 7 days of incubation in YEPD medium. Different lowercase letters denote significant differences at *P* = 0.05 based on one-way ANOVA test

Loss of BP1 function was previously shown to significantly alleviate transcriptional repression in *Fusarium graminearum*, especially for secondary metabolism-related genes [10]. The mycotoxin deoxynivalenol (DON) produced by *Fusarium* species is the most frequently detected specialized metabolite in cereal grains worldwide [25]. The regulation of DON biosynthesis is complex and has been extensively investigated [26]. We thus sought to determine here whether BP1 is necessary for the transcriptional repression of DON biosynthesis genes. Accordingly, we assembled the DON biosynthesis pathway based on multiple lines of evidence (Fig. 1A). Subsequent sequence, genetic, and biochemical analyses in *F. graminearum* identified three trichothecene biosynthesis (*TRI*) gene clusters consisting of 15 *TRI* genes encoding DON synthases [26]. Furthermore, we examined key *TRI* gene expression using previously published transcriptome deep sequencing (RNA-seq) data from *F. graminearum* loss of BP1 (Δ*BP1*) grown in yeast extract peptone dextrose (YEPD) medium, which does not induce toxin production (Fig. 1B). Reverse transcription quantitative PCR (RT-qPCR) of selected *TRI* genes in the strains Δ*BP1* and the wild-type PH-1 confirmed the RNA-seq results and revealed a significant upregulation of *TRI* gene expression in YEPD medium upon loss of BP1 function (Fig. 1C). We analyzed the enrichment of BP1 at key *TRI* loci using previously published BP1-GFP (green fluorescent protein) chromatin immunoprecipitation followed by sequencing (ChIP-seq) data (Fig. 1D). We also validated the enrichment of BP1 at these *TRI* loci using ChIP-quantitative real-time PCR (qPCR) upon growth in YEPD medium (Fig. 1E). As described previously, the toxisome is considered to be the compartment of DON biosynthesis in *F. graminearum* [25]. Tri1-GFP (a fusion between Tri1 and GFP) can be used as a visible marker to detect toxisome and DON formation. We therefore transformed the Tri1-GFP construct into the wild-type PH-1, Δ*BP1*, and Δ*BP1*-C strains. As shown in Fig. 1F, G, typical spherical toxisomes formed in Δ*BP1* but not in wild-type PH-1 or Δ*BP1*-C strains. Immunoblot analysis showed that Tri1-GFP strongly accumulated in the mycelia of the Δ*BP1* strain after 2 days of incubation on wheat (*Triticum aestivum*) leaves (Fig. 1H). In agreement, DON production was stronger in the Δ*BP1* strain than in the wild-type PH-1 and Δ*BP1*-C complementation strains in YEPD conditions (Fig. 1I). Taken together, these results suggest that BP1 suppresses DON biosynthesis in toxin non-inducing conditions.

### BP1 forms highly dynamic puncta in the nucleus

**Fig. 2:**
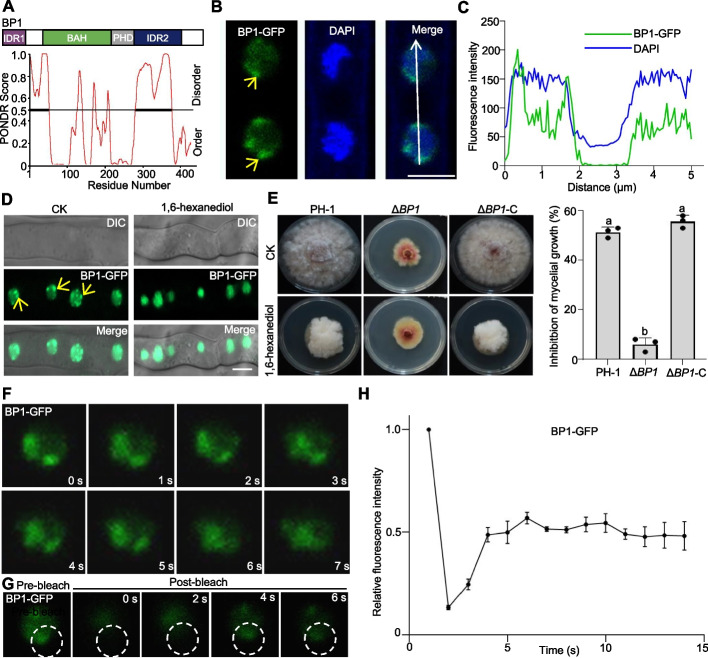
BP1 forms nuclear puncta in vivo. **A** Top, diagram of *F. graminearum* BP1 (top) with two intrinsically disordered regions (IDR1 and IDR2). Bottom, IDR analysis using the Predictor of Natural Disordered Regions (PONDR) database (http://pondr.com/). Regions with an average strength (PONDR score) ≥ 0.8 were considered to be disordered. **B** BP1 localization in hyphae of Δ*BP1*-C grown in YEPD medium at 25 °C for 24 h. The nuclei were stained with DAPI (4′,6-diamidino-2-phenylindole; blue fluorescence, 405-nm laser). The localization of BP1 to nuclear puncta is indicated by yellow arrows, scale bar, 2 μm. **C** Fluorescence intensity along the white line shown in **B** of the images of the Δ*BP1*-C strain expressing BP1-GFP and stained with DAPI. **D** BP1-GFP fluorescence in Δ*BP1*-C hyphae under control conditions (CK) or treated with 1% (w/v) 1,6-hexanediol before examination for GFP puncta. Scale bar, 5 μm. **E** 1,6-Hexanediol sensitivity of Δ*BP1*. The wild-type PH-1, Δ*BP1*, and Δ*BP1*-C strains were incubated on potato dextrose agar (PDA) containing 1% (w/v) 1,6-hexanediol for 3 days. Quantification of mycelial growth inhibition by 1,6-hexanediol for each strain is shown in the bar graph to the right. Different lowercase letters denote significant differences at *P* = 0.05. **F** Δ*BP1*-C complement strain grown in YEPD medium at 25 °C for 24 h. Fusion of nuclear puncta formed by BP1-GFP was examined by epifluorescence microscopy. Scale bar, 5 µm. **G** BP1-GFP nuclear puncta in the Δ*BP1* mutant were subjected to fluorescence recovery after photobleaching (FRAP) experiments using a Zeiss LSM 980 confocal laser-scanning microscope. The bleaching laser intensity was set to 50%, and the excitation wavelength was 488 nm. **H** Quantification of BP1-GFP fluorescence intensity before and after bleaching, with six droplets included in the analysis

Previous evidence revealed that BP1 recognized and directly bond to methylated H3K27 to facilitate transcriptional repression [10]. To examine the underlying regulatory mechanisms of BP1, especially related to DON biosynthesis, we analyzed the BP1 protein sequence, detecting a BAH domain, a PHD domain, and two IDRs (Fig. 2A, top). IDRs containing consecutive residues of the same or similar amino acids may form phase separation condensates [27]. Therefore, we investigated the phase separation potential of BP1 using the Predictor of Natural Disordered Regions (PONDR) database [28]. The PONDR results verified that BP1 contained two IDRs: IDR1 (N-terminal) and IDR2 (near its C terminus; Fig. 2A, bottom). Thus, bioinformatic analyses strongly support the presence of two IDRs in BP1, prompting us to focus on BP1 phase separation.

To explore the biological functions of BP1, we fused BP1 to GFP and drove the encoding construct by the *BP1* native promoter (*proBP1:BP1-GFP*). We transformed this construct into the BP1 deletion strain, generating the Δ*BP1-*C complementation strain, in which we observed the subcellular localization of BP1 in *F. graminearum*. As shown in Fig. 2B, C, we detected BP1-GFP in the nucleus, which we labeled using 4′,6-diamidino-2-phenylindole (DAPI). Notably, many puncta appeared in each nucleus, which led us to investigate the nature of these BP1 nuclear puncta. The aliphatic alcohol 1,6-hexanediol disrupts liquid–liquid phase-separated condensates [20]. We treated mycelia of the Δ*BP1*-C strain with 1% (w/v) 1,6-hexanediol. We determined that BP1-GFP puncta were dispersed and showed a diffuse nuclear localization after a 2-h 1,6-hexanediol treatment (Fig. 2D). Phenotypic analyses demonstrated that the Δ*BP1* strain was significantly less susceptible to 1,6-hexanediol compared to the wild-type PH-1 (Fig. 2E). We also observed dynamic fusion events between two adjacent BP1-GFP puncta in mycelia (Fig. 2F). Fluorescence recovery after photobleaching (FRAP) experiments revealed quick recovery of BP1-GFP puncta after targeted photobleaching (Fig. 2G, H), indicating the dynamic nature of BP1 puncta formation in the nucleus. These results suggest that BP1 nuclear puncta may undergo phase separation in vivo.

### BP1 undergoes liquid–liquid phase separation in vitro

**Fig. 3:**
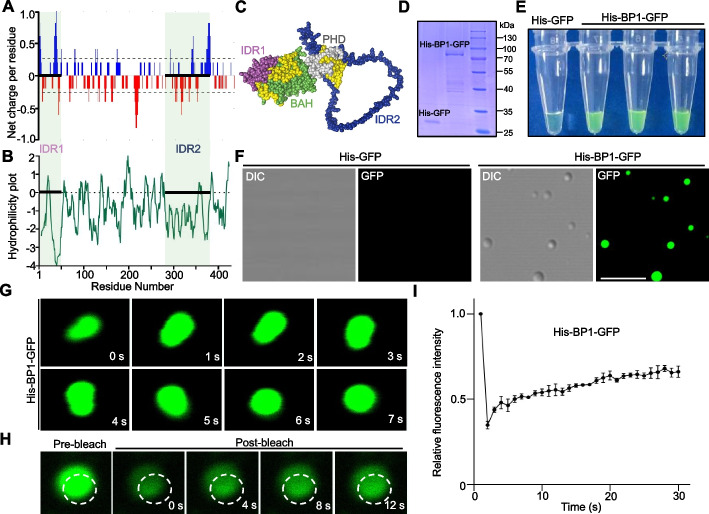
BP1 undergoes liquid–liquid phase separation in vitro. **A** Net charge per residue (NCPR) of BP1 was calculated using the Classification of Intrinsically Disordered Ensemble Regions (CIDER) web server (http://pappulab.wustl.edu/CIDER/analysis/). **B** Hydrophilicity plot of BP1 was predicted using ProtScale (https://web.expasy.org/protscale/). **C** 3D structure of BP1 was predicted using the AlphaFold Protein Structure Database (https://alphafold.ebi.ac.uk/). **D** Coomassie Brilliant Blue staining of recombinant His-GFP and His-BP1-GFP proteins purified from *E. coli*. **E** Turbidity visualization of recombinant His-GFP and His-BP1-GFP droplet formation. Tubes contained 30 μM His-GFP or His-BP1-GFP in 10% (w/v) PEG 8000 buffer. **F** Representative fluorescence and differential interference contrast (DIC) images of His-BP1-GFP droplets. Scale bar, 5 μm. **G** Confocal micrographs showing His-BP1-GFP droplets after phase separation and droplet fusion in vitro. **H** Phase-separated His-BP1-GFP droplets analyzed by fluorescence recovery after photobleaching (FRAP). The bleaching laser intensity was 100%, and representative images are shown. **I** Quantification of relative fluorescence recovery of His-BP1-GFP, six nuclei included in the analysis

IDRs form multivalent interactions based on electrostatic and/or hydrophobic interactions and play an important role in driving protein phase separation [18]. Thus, to determine if BP1 puncta were liquid droplets, we analyzed the net charge per residue, hydrophilicity, and tertiary structure of BP1. We determine that IDR1 and IDR2 harbor many positively charged residues and are completely hydrophilic (Fig. 3A, B). Furthermore, we predict the three-dimensional structure of BP1 by the AlphaFold Protein Structure Database. AlphaFold produces a per-residue confidence metric called the predicted local distance difference test (pLDDT), yielding a scale from 0 to 100 [29]. Some regions below 50 pLDDT may be unstructured in isolation [30]. Notably, the average pLDDT scores of the IDRs were 60.4 (IDR1) and 37.5 (IDR2). According to the structural analysis, IDR2 is more exposed to the environment than the other structural domains of BP1 (Fig. 3C). Many IDR-containing proteins form dynamic liquid-like droplets or gel-like phase-separated condensates in vitro due to multivalent and weak interactions among IDRs [31]. We thus evaluated phase separation in vitro by assessing fluorescent droplet formation and solution turbidity [24]. To examine whether BP1 formed liquid-like droplets in vitro, we purified recombinant His-BP1-GFP from *Escherichia coli* (Fig. 3D). Recombinant His-BP1-GFP was soluble and did not form droplets under physiological salt conditions. We then tested the effect of 10% (w/v) polyethylene glycol 8000 (PEG 8000), which induces a crowding effect that triggers protein phase separation. When we added 10% PEG 8000 to the His-BP1-GFP protein solution, we observed a clear change to turbidity (Fig. 3E). We also performed protein phase separation assays with increasing concentrations of His-BP1-GFP, ranging from 5 to 30 μM. As shown in Additional file 1: Fig. S1A, B, BP1 droplet formation was concentration dependent. The number and size of droplets increased with increasing protein concentrations. We noticed the formation of the greatest number and largest droplets with 30 μM His-BP1-GFP. Fluorescence microscopy detected many round, fluorescent droplets in the turbid His-BP1-GFP solution (Fig. 3F). Under the same conditions, His-GFP did not form droplets (Additional file 1: Fig. S2A). Importantly, His-BP1-GFP droplets were sensitive to treatment with 1% 1,6-hexanediol (Additional file 1: Fig. S2A). We observed that two His-BP1-GFP droplets readily fused into a larger droplet once they made contact (Fig. 3G). In addition, FRAP experiments showed that His-BP1-GFP within droplets dynamically exchanged with His-BP1-GFP in the surrounding environment after intense laser bleaching (Fig. 3H, I). To exclude the possibility of interference by the GFP tag in the phase separation assays, we purified His-BP1 from *E. coli* (Additional file 1: Fig. S3A). Under the same phase separation assay conditions as His-BP1-GFP, His-BP1 also showed LLPS properties and formed phase-separated droplets (Additional file 1: Fig. S3B-E). Thus, our results demonstrate that BP1 undergoes LLPS to form liquid droplets in vitro.

### IDR2 mediates BP1 phase separation in vitro and in vivo

**Fig. 4:**
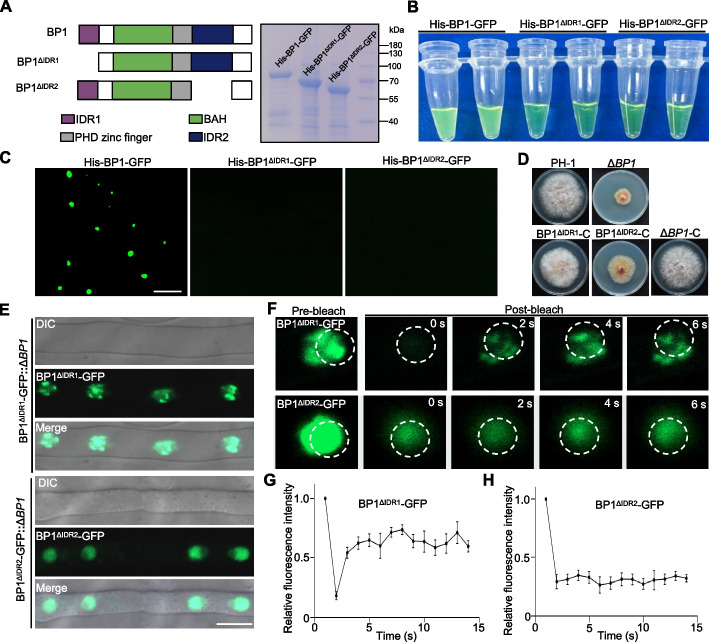
IDR2-dependent BP1 phase separation is critical for its localization to nuclear puncta. **A** Diagrams of IDR-deletion BP1 variants (left panel). Coomassie Brilliant Blue staining of recombinant His-BP1-GFP, His-BP1^ΔIDR1^-GFP, and His-BP1^ΔIDR2^-GFP proteins purified from *E. coli* (right panel). **B** Assessment of turbidity indicating recombinant His-BP1-GFP, His-BP1^ΔIDR1^-GFP, and His-BP1^ΔIDR2^-GFP droplet formation. Tubes contained 30 μM His-BP1-GFP, His-BP1^ΔIDR1^-GFP, or His-BP1^ΔIDR2^-GFP in 10% (w/v) PEG 8000. **C** Representative fluorescence images of His-BP1-GFP, His-BP1^ΔIDR1^-GFP, and His-BP1^ΔIDR2^-GFP droplets. Scale bar, 5 μm. **D** Images showing the vegetative growth phenotypes of wild-type PH-1, Δ*BP1*, and the complementation strains Δ*BP1*-C, BP1^ΔIDR1^-C (lacking IDR1), and BP1^ΔIDR2^-C (lacking IDR2), grown on PDA medium for 3 days before imaging. **E** Subcellular localization of the IDR-deletion variants, BP1^ΔIDR1^-C, and BP1^ΔIDR2^-C, in mycelia grown in YEPD medium for 24 h. Scale bar, 5 µm. **F** BP1^ΔIDR1^-GFP nuclear puncta (upper panels) and BP1^ΔIDR2^-GFP diffuse nuclear localization (lower panels were subjected to FRAP experiments using a Zeiss LSM 980 confocal laser-scanning microscope). The bleaching laser intensity was set to 50%, and the excitation wavelength was 488 nm. **G, H** Quantification of BP1^ΔIDR1^-GFP (**G**) and BP1^ΔIDR2^-GFP (**H**) fluorescence intensity before and after bleaching, with six nuclei analyzed per strain

Since BP1 contains two IDRs, IDR1 and IDR2, we wondered which IDR was the key region influencing the phase separation of BP1. Accordingly, we generated constructs encoding the truncated proteins His-BP1^ΔIDR1^-GFP and His-BP1^ΔIDR2^-GFP, lacking either IDR1 or IDR2, which we purified from *E. coli* (Fig. 4A). The truncated variants His-BP1^ΔIDR1^-GFP and His-BP1^ΔIDR2^-GFP were soluble and did not form droplets (Fig. 4B, C). Furthermore, we generated *His-IDR1-GFP* and *His-IDR2-GFP* constructs to purify the recombinant proteins from *E. coli* (Additional file 1: Fig. S4A). We evaluated droplet formation of His-IDR1-GFP and His-IDR2-GFP. As shown in Additional file 1: Fig. S4B, the His-IDR1-GFP and His-IDR2-GFP solutions immediately became turbid after the addition of 10% PEG 8000. Microscopy observations showed that His-IDR1-GFP and His-IDR2-GFP formed many round, fluorescent droplets in the turbid solution (Additional file 1: Fig. S4C). His-IDR1-GFP and His-IDR2-GFP droplets were also sensitive to 1,6-hexanediol (Additional file 1: Fig. S2B, C). These results indicate that the two IDRs of BP1 determine phase separation in vitro.

To further investigate the role of the two BP1 IDRs in vivo, we cloned the *BP1* open reading frame lacking either IDR (*BP1*^*ΔIDR1*^ or *BP1*^*ΔIDR2*^) in-frame and upstream of *GFP* under the control of the native *BP1* promoter. We then individually introduced the resulting *BP1*^*ΔIDR1*^*-GFP* and *BP1*^*ΔIDR2*^*-GFP* cassettes into the Δ*BP1* strain, generating the complementation strains BP1^ΔIDR1^-C and BP1^ΔIDR2^-C (Fig. 4D). We characterized the complementation strains by PCR amplification, RT-qPCR, and immunoblot assays (Additional file 1: Fig. S5) before evaluating their phenotypes. Surprisingly, the BP1^ΔIDR1^-C strain showed a growth phenotype similar to that of wild-type PH-1. The *BP1pro:BP1*^*ΔIDR2*^*-GFP* construct failed to complement the growth defects of the Δ*BP1* strain (Fig. 4D and Additional file 1: Fig. S1C). These results indicate that IDR2 is important for BP1 function in *F. graminearum* vegetative growth. We asked whether BP1^ΔIDR1^-GFP and/or BP1^ΔIDR2^-GFP showed altered localization patterns in the nucleus. Confocal microscopy observation of BP1^ΔIDR1^-GFP and BP1^ΔIDR2^-GFP localization indicated that BP1^ΔIDR2^-GFP exhibited a diffuse nuclear localization in mycelia, lacking an obvious punctate pattern. By contrast, BP1^ΔIDR1^-GFP formed many nuclear puncta (Fig. 4E and Additional file 1: Fig. S1D). Furthermore, BP1^ΔIDR1^-GFP localization in mycelia showed a diffuse nuclear localization after a 2-h 1,6-hexanediol treatment (Additional file 1: Fig. S2D). The diffuse nuclear localization of BP1^ΔIDR2^-GFP was no change after a 2-h 1,6-hexanediol treatment (Additional file 1: Fig. S2E). In addition, FRAP experiments showed that BP1^ΔIDR1^-GFP nuclear puncta dynamically exchanged with BP1^ΔIDR1^-GFP in the surrounding environment after intense laser bleaching (Fig. 4F upper panels, G). BP1^ΔIDR2^-GFP in the Δ*BP1* background showed no change in its subcellular distribution following intense laser bleaching (Fig. 4F lower panels, H). Taken together, these results demonstrate that IDR2 is a key region driving the phase separation of BP1 in the nucleus.

### IDR2 of BP1 is critical for PRC2 interaction and H3K27me3 binding

**Fig. 5:**
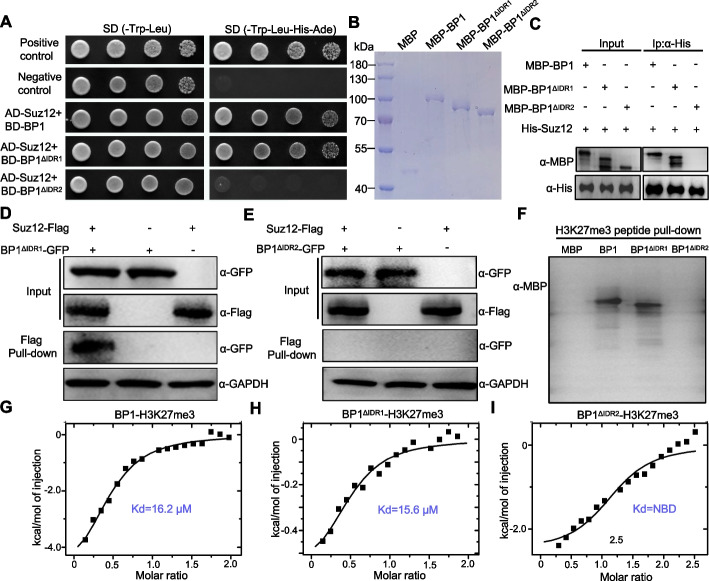
IDR2 of BP1 drives PRC2 interaction and H3K27me3 binding. **A** BP1 interaction with Suz12, a component of PRC2, requires IDR2 of BP1 in a yeast two-hybrid (Y2H) assay. Yeast cells harboring the indicated bait and prey constructs were assayed for growth on synthetic defined (SD) medium lacking leucine, tryptophan, histidine, and adenine SD (–Leu–Trp–His–Ade). The plasmid pair pGBKT7-53 and pGADT7 was used as the positive control. Another pair of plasmids, pGBKT7-Lam and pGADT7, was used as the negative control. Images were taken after 3 days of incubation at 30 °C. **B** SDS-PAGE analysis of recombinant MBP-BP1 and BP1 IDR deletion variants (MBP-BP1^ΔIDR1^ and MBP-BP1^ΔIDR2^) purified from *E. coli*. **C** Pull-down assay of the interaction between MBP-BP1^ΔIDR1^ or MBP-BP1^ΔIDR2^ and His-Suz12. Full-length MBP-BP1 was used as a positive control. **D**, **E** Interaction between BP1^ΔIDR1^-GFP (**D**), BP1^ΔIDR2^-GFP (**E**), and Suz12-Flag as examined by co-immunoprecipitation (Co-IP) in *F. graminearum*. GAPDH used as the loading control. **F** Peptide pull-down assays using the H3K27me3 peptide and either recombinant MBP-BP1 or its deletion variants (MBP-BP1^ΔIDR1^ and MBP-BP1^ΔIDR2^). MBP served as a negative control. Immunoprecipitation by biotinylated H3K27me3 was analyzed with an anti-MBP antibody. **G**–**I** Isothermal titration calorimetry (ITC) assays performed to measure the binding affinity of MBP-BP1 (**G**), MBP-BP1^ΔIDR1^ (**H**), or MBP-BP1^ΔIDR2^ (**I**) to the H3K27me3 peptide. NDB, no detectable binding

BP1 interacts with the core PRC2 complex component Suz12 and binds to H3K27me3 to reinforce transcriptional repression of H3K27me3-decorated genes [10]. To determine whether BP1 IDRs participate in the BP1–Suz12 interaction, we used the deletion variants BP1^ΔIDR1^ and BP1^ΔIDR2^ in yeast two-hybrid (Y2H) assays. We established that IDR2 was essential for the BP1–Suz12 interaction in yeast (Fig. 5A). To further test the interaction between BP1 and Suz12 in vitro, we produced maltose-binding protein (MBP)-tagged BP1, BP1^ΔIDR1^, and BP1^ΔIDR2^ as well as His-tagged Suz12 in *E. coli* and performed pull-down assays (Fig. 5B). Both BP1 and BP1^ΔIDR1^ immunoprecipitated with Suz12 under these conditions, but we detected no interaction between BP1^ΔIDR2^ and Suz12 (Fig. 5C). To test whether the IDR is necessary for the BP1–Suz12 interaction in vivo, we performed a co-immunoprecipitation (Co-IP) assay in the wild-type strain carrying *BP1*^*ΔIDR1*^*-GFP* or *BP1*^*ΔIDR2*^*-GFP* and the *Suz12-Flag* constructs. As shown in Fig. 5D, BP1 lacking IDR1 still interacted with Suz12. However, BP1 lacking IDR2 was unable to interact with Suz12 (Fig. 5E). Taken together, these results indicate that IDR2 is essential for the BP1–PRC2 interaction in *F. graminearum*.

Given that BP1 is a H3K27me3 reader, we investigated whether loss of IDR1 or IDR2 affected BP1 ability to bind to H3K27me3. To this end, we performed a histone peptide pull-down assay using recombinant MBP-tagged BP1, BP1^ΔIDR1^, and BP1^ΔIDR2^. Consistent with previous findings, MBP-BP1 was pulled down by the H3K27me3 peptide [10]. MBP-BP1^ΔIDR1^ also displayed strong binding to H3K27me3, whereas MBP-BP1^ΔIDR2^ could not be pulled down by H3K27me3 (Fig. 5F). We next tested whether BP1 lacking IDR1 or IDR2 bound H3K27me3 by isothermal titration calorimetry (ITC) assay. Consistent with the results of the histone peptide pull-down assays, ITC measurements of H3K27me3 revealed a dissociation constant (Kd) of 16.2 μM for BP1 (Fig. 5G) and 15.6 μM for BP1^ΔIDR1^ (Fig. 5H), while we observed no binding for BP1^ΔIDR2^ (Fig. 5I). These results indicate that IDR2 of BP1 is responsible for specific recognition of H3K27me3. Together, these data support a previously uncharacterized role for BP1 LLPS in regulating the interaction between BP1, PRC2, and the recognition of H3K27me3.

### BP1 phase separation mediates H3K27me3 target gene silencing

**Fig. 6:**
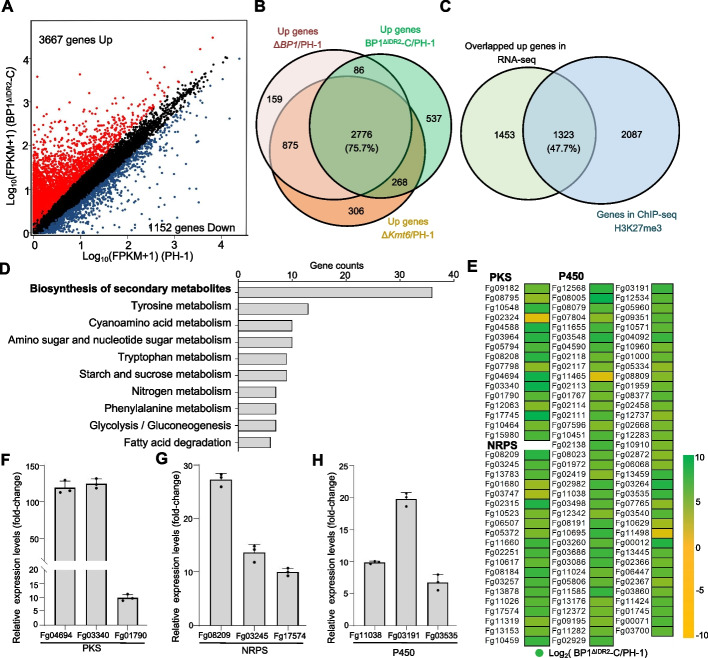
IDR2 of BP1 is required for H3K27me3-mediated transcriptional repression of secondary metabolite–related genes. **A** Scatterplot of differentially expressed genes between the wild-type PH-1 and BP1^ΔIDR2^-C *F. graminearum* as identified by RNA-seq. The red circles represent significantly upregulated genes, and blue circles represent significantly downregulated genes (*P* value ≤ 0.05 and fold-change ≥ 2 or ≤  − 2, respectively). Genes not differentially expressed are shown in black. **B** Venn diagram showing the overlap between significantly upregulated genes in BP1^ΔIDR2^-C, Δ*BP1*, and Δ*Kmt6*. **C** Venn diagram showing the overlap between significantly upregulated genes in BP1^ΔIDR2^-C, Δ*BP1*, and Δ*Kmt6* strains and genes harboring H3K27me3 (as identified by chromatin immunoprecipitation sequencing, ChIP-seq). **D** Kyoto Encyclopedia of Genes and Genomes (KEGG) analysis of the overlapping upregulated, H3K27me3-enriched genes in panel C. The top 10 significantly pathways are listed, based on enriched gene counts. **E** Heatmap representation of the transcriptional changes of selected secondary metabolism biosynthesis genes in BP1^ΔIDR2^-C strain compared to those in wild-type *F. graminearum*. Selected genes included those encoding polyketide synthases (PKSs), non-ribosomal peptide synthases (NRPSs), and cytochrome P450 enzymes. **F**–**H** Transcript levels of secondary metabolism biosynthesis genes in BP1^ΔIDR2^-C as determined by RT-qPCR. Relative transcript levels were normalized to *ACTIN* as the internal standard and presented as means ± SD from three independent experiments. Different lowercase letters denote significant differences at *P* = 0.05 based on one-way ANOVA test

Previous genetic analyses suggested that BP1 was involved in H3K27me3-mediated gene repression. To determine the effect of LLPS on BP1 function in vivo, we investigated the molecular phenotype of the BP1^ΔIDR2^-C strain through RNA-seq analysis. Similar to the transcriptional patterns seen in the Δ*BP1* strain, we identified 3667 upregulated genes and 1152 downregulated genes in BP1^ΔIDR2^-C (*P* value ≤ 0.05, fold-change ≥ 2 or fold-change ≤  − 2; Fig. 6A; Additional file 2: Table S1). We recently described Kmt6 as a writer and BP1 as a reader of H3K27me3 in *F. graminearum* [10]. To determine whether BP1 LLPS regulates H3K27me3 target gene repression, we compared the BP1^ΔIDR2^-C transcriptome generated above to RNA-seq data for the Δ*Kmt6* and Δ*BP1* mutants. Many genes (2776; representing 75.7% of all upregulated genes in BP1^ΔIDR2^-C) were upregulated in the Δ*Kmt6*, Δ*BP1*, and BP1^ΔIDR2^-C strains (Fig. 6B; Additional file 2: Table S2). We reanalyzed whether these co-upregulated genes (Fig. 5B) were associated with the H3K27me3 mark using our previously generated H3K27me3 ChIP-seq data [10]. Among these co-upregulated genes, we identified 1323 genes (47.7% of the co-upregulated genes among Δ*Kmt6*, Δ*BP1*, and BP1^ΔIDR2^-C) marked by H3K27me3 (Fig. 6C; Additional file 2: Table S3). These results indicate that BP1 phase separation is required for H3K27me3-regulated transcriptional repression.

H3K27me3 plays important roles in secondary metabolite biosynthesis and is considered to be a repressor of secondary metabolism [11]. We thus investigated the role of BP1 IDR2 in the biosynthesis of secondary metabolites. Kyoto Encyclopedia of Genes and Genomes (KEGG) pathway analysis revealed that genes overlapping in Fig. 6C were significantly enriched in secondary metabolite biosynthesis pathways (Fig. 6D). We specifically examined *F. graminearum* secondary metabolism genes encoding polyketide synthases (PKSs), non-ribosomal peptide synthases (NRPSs), and cytochrome P450 enzymes and visualized the expression profiles in BP1^ΔIDR2^-C strain using heatmaps. We observed that 71.6% (78/109) of all selected genes were significantly upregulated in BP1^ΔIDR2^-C compared to those in the wild type (*P* value ≤ 0.05), showing almost the same expression patterns as in the Δ*BP1* strain (Fig. 6E; Additional file 2: Table S4). We also confirmed the differential expression of several secondary metabolism genes by RT-qPCR in the BP1^ΔIDR2^-C strain (Fig. 6F, H). These data further demonstrate that phase separation of BP1 is required for H3K27me3-mediated transcriptional repression, especially for secondary metabolism genes.

### IDR2 is important for BP1 function in DON production and pathogenesis

**Fig. 7:**
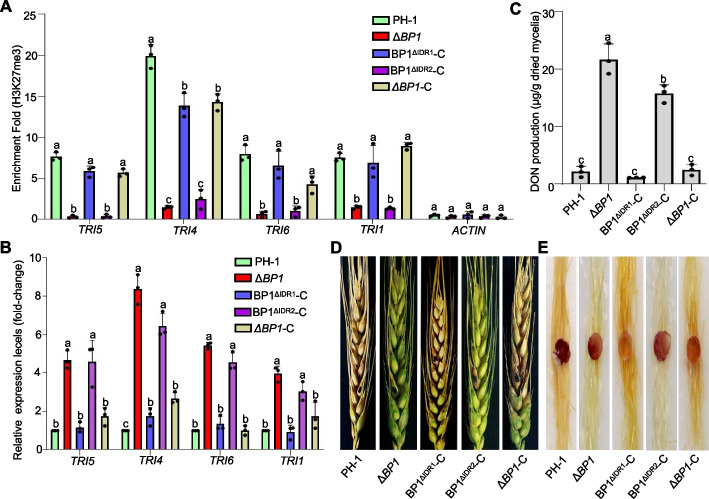
BP1^ΔIDR2^-C shows increased DON production and attenuated virulence *in planta*. **A** ChIP-qPCR analysis of relative H3K27me3 levels at *TRI* genes (*TRI5*, *TRI4*, *TRI6*, and *TRI1*) in wild-type PH-1, Δ*BP1*, BP1^ΔIDR1^-C, BP1^ΔIDR2^-C, and Δ*BP1*-C *F. graminearum* strains grown in toxin non-inducing conditions (YEPD) for 48 h. *ACTIN* served as a negative control. **B** Wild-type PH-1, Δ*BP1*, BP1^ΔIDR1^-C, BP1^ΔIDR2^-C, and Δ*BP1*-C strains were cultured in YEPD medium for 48 h. Total RNA was extracted for RT-qPCR analysis to determine relative transcript levels of *TRI* genes. Transcript levels were normalized to *ACTIN*. **C** DON production in the wild-type, Δ*BP1* mutant, and complementation strains (BP1^ΔIDR1^-C, BP1^ΔIDR2^-C and Δ*BP1*-C) after 7 days of growth in YEPD medium. Different lowercase letters denote significant differences at *P* = 0.05 based on one-way ANOVA test. **D**, **E** Pathogenicity of the wild-type, mutant, and various complementation strains incubated in the central section spikelet of single flowering wheat head for 15 days (**D**) and on maize silks for 5 days (**E**)

DON is a large family of sesquiterpenoid secondary metabolites and an important virulence factor in *F. graminearum* [32]. To gain insights into the role of BP1 phase separation in *TRI* transcriptional repression, we examined the enrichment of H3K27me3 at *TRI* loci. To this end, we performed a ChIP-qPCR analysis using an anti-H3K27me3 antibody under different conditions. As shown in Additional file 1: Fig. S5A, we detected a strong drop in H3K27me3 enrichment at the selected genes (*TRI5*, *TRI4*, *TRI6*, and *TRI1*) in toxin-inducing conditions (TBI), compared to those in toxin non-inducing conditions (YEPD medium). We conducted an RT-qPCR analysis to assess the relative transcript levels of *TRI* genes in strains grown in YEPD or TBI conditions. We detected lower H3K27me3 enrichment at *TRI* loci under TBI conditions compared to YEPD conditions (Additional file 1: Fig. S5A), leading to increased *TRI* gene expression (Additional file 1: Fig. S5B), consistent with previous observations in plants and animals that reduced H3K27me3 enrichment tends to result in transcriptional de-repression [33]. These results indicate that *TRI* gene expression is associated with H3K27me3 enrichment. To determine whether BP1 phase separation contributes to the repression of DON biosynthesis genes, we compared H3K27me3 enrichment at *TRI* genes in wild-type PH-1, Δ*BP1*, BP1^ΔIDR1^-C, BP1^ΔIDR2^-C, and Δ*BP1*-C strains by ChIP-qPCR. As in the Δ*BP1* mutant, BP1^ΔIDR2^-C exhibited a reduction in H3K27me3 enrichment at *TRI* genes compared to those in the PH-1, BP1^ΔIDR1^-C, and Δ*BP1*-C strains (Fig. 7A). To test whether the reduction in H3K27me3 enrichment at the *TRI* genes resulted in transcript accumulation, we performed RT-qPCR analysis. Indeed, we observed an upregulation of the selected *TRI* genes in the Δ*BP1* and BP1^ΔIDR2^-C strains relative to the wild type and BP1^ΔIDR1^-C (Fig. 7B). Consistent with the RT-qPCR results above, DON accumulation was significantly higher in the Δ*BP1* and BP1^ΔIDR2^-C strains compared to wild-type PH-1, BP1^ΔIDR1^-C and Δ*BP1*-C strains (Fig. 7C). To investigate the function of BP1 phase separation in pathogenicity, we evaluated the virulence of the wild-type and mutant strains in the flowering wheat heads and maize (*Zea mays*) silks. BP1^ΔIDR2^ could not rescue the attenuated virulence of Δ*BP1*, whereas the wild-type PH-1, BP1^ΔIDR1^-C, and Δ*BP1*-C strains quickly spread across the flowering wheat heads and maize silk (Fig. 7D, E). Together, these results suggest that BP1 phase separation affects gene expression, at least partially, by stabilizing H3K27me3 deposition at target genes, especially *TRI* genes.

### Phase separation of BP1 orthologs is conserved in fungi

**Fig. 8:**
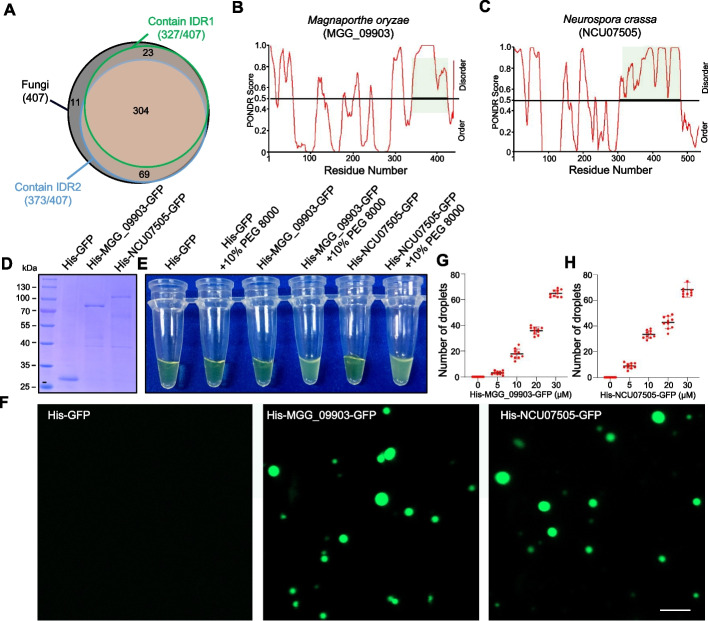
Phase separation of BP1 orthologs is ubiquitous in fungi. **A** Venn diagram of IDR distribution in BP1 orthologs from fungi (407 total, black). Among the fungal BP1 orthologs, the number of proteins containing IDR1 (327, green) and IDR2 (373, blue) is shown. **B**, **C** The IDRs of *Magnaporthe oryzae* MGG_09903 (**B**) and *Neurospora crassa* NCU07505 (**C**) were detected by the Predictor of Natural Disordered Regions database. **D** Coomassie brilliant blue staining of recombinant His-MGG_09903-GFP and His-NCU07505-GFP proteins purified from *E. coli*. **E** Turbidity assay of recombinant His-MGG_09903-GFP and His-NCU07505-GFP in buffer containing 10% (w/v) PEG 8000 to visualize separation of high-concentration condensates. **F** Representative micrographs of His-MGG_09903-GFP and His-NCU07505-GFP protein preparations. **G**, **H** Quantifications of droplet numbers (**G**) and droplet area (**H**) are shown

BP1 orthologs are widespread in fungi. We asked whether phase separation is conserved among BP1 orthologs in various fungi by inspecting 407 fungal genomes encoding BP1 orthologs [10]. We performed IDR structural analysis using the PONDR database. As shown in Fig. 8A, the protein sequences of most BP1 homologs contained IDR1 or IDR2 (97.3%, 396 of 407 genes, average strength ≥ 0.8). Notably, we observed that 91.65% of the tested BP1 homologs (373 of 407 genes, average strength ≥ 0.8) had a clear IDR2, demonstrating that IDR2 was conserved across microbial taxa. We identified *Magnaporthe oryzae* MGG_09903 and *Neurospora crassa* NCU07505 as encoding BP1 orthologs and detected a clear IDR2 near their C termini (Fig. 8B, C). We explored whether the IDR2-containing proteins encoded by MGG_09903 and NCU07505 might form droplet-like condensates by LLPS using purified recombinant His-MGG_09903-GFP and His-NCU07505-GFP from *E. coli* (Fig. 8D). When 10% PEG 8000 was added to the His-MGG_09903-GFP and His-NCU07505-GFP protein solutions, the clear solutions quickly became turbid (Fig. 8E). We observed micrometer-sized droplets by confocal microscopy (Fig. 8F). The number of His-MGG_09903-GFP or His-NCU07505-GFP droplets increased as protein concentration increased (Fig. 8G, H). These results indicate that His-MGG_09903-GFP and His-NCU07505-GFP undergo LLPS, suggesting that phase separation of BP1 orthologs occurs in various fungal species.

## Discussion

Histone PTMs are a dynamic and reversible gene regulatory mechanism conserved among animals, plants, and fungi [34]. Recent studies have reported a multitude of reader proteins with diverse functions and mechanisms in animals and plants [35]. Histone readers recognize histone PTMs in a modification state- and position-dependent manner to alter chromatin structure or recruit other chromatin-related factors [36]. Over the past decade, although versatile reader proteins have been identified and characterized, some fundamental questions remain to be systematically addressed, especially regarding the role of readers in regulating gene expression.

In human and *Drosophila*, HP1 binds to the histone H3 mark lysine 9 methylation (H3K9me) and forms phase-separated droplets to facilitate heterochromatin-mediated gene silencing [37]. Swi6, the HP1 homolog in fission yeast (*Schizosaccharomyces pombe*), exhibits dynamics that are characteristic of LLPS to mediate chromatin compaction [38]. Human MeCP2 (methyl-CpG binding protein 2) is a transcriptional repressor that interacts with methylated DNA and H3K27me3 in neurons [39]. Recent results provide a new perspective on the mechanisms by which LLPS drives the MeCP2-mediated formation of distinct heterochromatin foci [21, 40]. Furthermore, crystal structures have revealed that the epigenetic reader protein ZMYND8 recognizes dual histone marks, H3K4 methylation, and H3K14 acetylation [5, 41]. ZMYND8 forms phase-separated liquid compartments to suppress metastasis-related gene expression [23]. Inspired by the reader proteins of animals and plants, here we demonstrate that BP1, a H3K27me3 reader in fungi, forms phase-separated liquid condensates through LLPS in vivo and in vitro (Figs. 2 and 3). Our data, together with those of previously published studies, provide several lines of evidence to reveal a common, yet important, role for histone reader proteins in regulating transcription through LLPS. However, clustering of BP1 protein did not form droplets on its own. Recent studies have shown that the formation of phase-separated condensates of CBX2-PRC1 can be due to either as drivers or clients through a concentration- and composition-dependent scaffold-client model [42]. The clustering of BP1 protein may also be associated with its interacting protein. Therefore, the molecular mechanisms underlying BP1 clustering needs to further clarify in the future.

Biochemical and cytological experiments have enabled the identification and functional analysis of protein phase separation. Exploring how LLPS of histone reader proteins dynamically regulates gene expression is fundamental to understanding many biological events in various organisms. LLPS of histone reader proteins in animals and plants has received significant attention as a mechanism for the rapid and transient assembly of condensed phase droplets to precisely regulate gene expression in time and space [43, 44]. In this study, IDR deletion assays demonstrate that LLPS of BP1 regulates the interaction of BP1 with Suz12, a component of the PRC2 complex (Fig. 5A–C), which deposits H3K27me3 at target genes to mediate their silencing [33]. Thus, we used the deletion variants BP1^ΔIDR1^ and BP1^ΔIDR2^ to detect binding to the H3K27me3 peptide. We established that IDR2 is important for H3K27me3 recognition (Fig. 5D–F). The molecular mechanisms underlying the function of BP1 LLPS may be two-fold. First, these puncta showed properties of LLPS by stabilizing BP1 localization to nuclear puncta in an IDR2-dependent way. Second, BP1 LLPS might regulate the interaction of BP1 with the PRC2 complex and facilitate the recognition of H3K27me3 to maintain transcriptional repression.

Fungi produce diverse low-molecular-weight compounds known as secondary metabolites (SMs). SMs have critical roles in transcription, development, and plant–fungal pathogen interactions [45]. Recent studies have revealed that genes involved in fungal SM biosynthesis are clustered and often located close to telomeres [46]. Enzymes for SM biosynthesis are contained within conserved subcellular compartments and localize to particular organelles to promote biosynthetic efficiency [47]. *F. graminearum* is a species-rich group of mycotoxigenic plant pathogens that ranks as the most widespread in cereal crops worldwide [48]. DON mycotoxin is a large family of SMs produced by a host of *Fusarium* species and causes adverse health effects in humans and livestock [26]. The biosynthetic enzymes required for DON production are encoded by 15 *TRI* genes, which are located in three gene clusters [49]. In addition, the enzymes catalyzing DON biosynthesis localize to spherical structures [50]. More recently, these cellular structures were identified as reorganization of the endoplasmic reticulum and named “toxisomes” [51]. Consistent with the protein phase separation that underlies the formation of intracellular compartments, compartmentalization of SM biosynthetic pathways, including biosynthetic enzymes and sites, controls fungal SM biosynthesis. However, studies linking LLPS to SM biosynthesis are lacking. Here, we showed here that SM biosynthesis genes encoding PKS, NRPS, and cytochrome P450 enzymes are significantly upregulated in the BP1^ΔIDR*2*^-C strain. In agreement with the H3K27me3-mediated regulation of fungal SM biosynthesis in different fungi [52], we demonstrated that IDR2 of BP1 regulated H3K27me3 enrichment at *TRI* loci to maintain their transcriptional repression in toxin non-inducing YEPD medium. We suggest that LLPS of BP1 increases H3K27me3 deposition at *TRI* loci to reinforce transcriptional repression. This work highlights the importance of protein phase separation in regulating SM biosynthesis and has implications for the long-standing controversial notion that SM genes are silenced in fungi.

Proteins that undergo LLPS in vitro and in vivo often contain IDRs that mediate biomolecular condensate formation [53]. IDR1 and IDR2 of BP1 both underwent LLPS to form droplets in vitro (Additional file 1: Fig. S4). Unexpectedly, only IDR2 was a key region for BP1 nuclear puncta localization in vivo and had phase separation potential (Fig. 4E, F). Previous studies indicated that deleting IDRs altered the subcellular localization of their respective proteins [18, 20]. Consistent with this result, the *BP1pro:BP1*^*ΔIDR2*^ construct failed to rescue the growth defect of Δ*BP1* (Fig. 3K). These results provide direct experimental evidence that IDR2 drives BP1 liquid condensate formation, which is required for its function. PONDR database analyses showed that IDR2 is conserved among BP1 homologs from various fungi (Fig. 8A-C). *M. oryzae* MGG_09903 and *N. crassa* NCU07505, two orthologous of BP1, exhibited LLPS properties (Fig. 8F). Taken together, our findings indicate that phase separation of BP1 orthologs is widespread in fungi and lead to reinterpretation of how histone readers function in sustaining gene expression. We envision that our studies will pave the way for future work focusing on the role of histone PTM phase separation in transcriptional regulation.

**Fig. 9:**
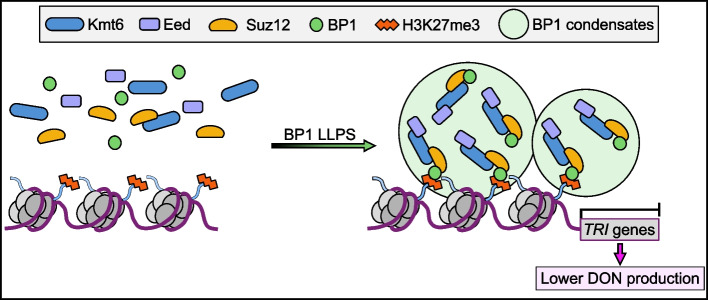
A proposed model showing the role of BP1 phase separation in maintaining the transcriptional repression of DON mycotoxin biosynthesis genes. Under normal growth condition, H3K27me3 reader BP1 undergoes LLPS to form dynamic phase-separated liquid condensates in the nucleus. The condensates recruit and concentrate PRC2 protein complex (Kmt6-Suz12-Eed) to effectively regulate transcriptional repression of H3K27me3 binding genes, such as DON mycotoxin biosynthesis *TRI* genes, leading lower DON production

Gene expression is generally discontinuous, consisting of transcriptional initiation and termination at timescales ranging from seconds to minutes [54]. Although the molecular mechanisms involved in transcriptional regulation have been studied extensively, there is little consensus in live cells. Over the past decade, numerous studies have indicated that the potential reversibility of histone PTMs has a significant influence on dynamic gene expression [55]. Recently, the umbrella term “transcriptional bursting” has been employed to explain a range of potentially different phenomena and has received considerable interest [56]. LLPS, an energy-saving mechanism that can form dynamic clusters in live cells resembling biomolecular condensates, likely enables cooperative and robust transcriptional bursting [57, 58]. Emerging evidence also suggests that LLPS can modulate burst properties to control transcriptional activation of target genes [59]. Transcriptional activation has been relatively well studied in eukaryotes. Here, we demonstrate that LLPS of histone reader proteins underlies transcriptional repression, which has important implications for secondary metabolite-related gene repression, especially DON mycotoxin biosynthesis genes in *F. graminearum* (Fig. 9). Thus, LLPS is a double-edged sword that positively and negatively affects transcriptional activity. In future studies, it will be important to consider the effects of LLPS in various physiological programs, including signal transduction and plant–pathogen interactions. Existing evidence provides a conceptual framework that small molecules, which modulate LLPS, can be used as a potential treatment for cancer [60]. Unlike canonical drug targets, directly targeting LLPS events may be a new therapeutic approach in cancer [61]. Therefore, it is plausible that manipulation of phase separation may represent a promising strategy for the management of mycotoxins in fungi, a possibility that merits further study.

## Conclusion

In summary, we elucidate that BP1 dynamically forms phase-separated liquid condensates to modulate its biological function. Deletion and replacement assays identify that IDR2 of BP1 is responsible for its LLPS. Furthermore, loss of IDR2 abolishes the binding activity of BP1 to H3K27me3 and relieve DON mycotoxin biosynthesis *TRI* genes transcription repression. These results reveal that the histone reader BP1 contributes to the maintenance of H3K27me3 regulating gene transcription repression by forming liquid–liquid phase separation condensates. Overall, we report a new mechanism for histone reader BP1-mediated gene transcription repression in fungi. This study advances a conceptual framework for studies of functional roles of histone modification in transcriptional regulation.

## Methods

### Fungal strains and cultivation conditions

*Fusarium graminearum* strain PH-1 (NRRL 31084) was used as a parental strain for the deletion experiments in this study. All strains were maintained on potato dextrose agar (PDA; 200 g potato, 20 g glucose, 10 g agar, and 1 L water) medium and incubated at 25 °C under a 12-h-light/12-h-dark cycle to examine growth rate. To assess the susceptibility of wild-type PH-1 and mutant strains to the LLPS disturbing agent 1,6-hexanediol, mycelial agar plugs were placed on PDA medium alone or with 1% 1,6-hexanediol and incubated for 72 h. Mycelia grown in liquid YEPD medium (10 g peptone, 3 g yeast extract, 20 g D-glucose, and 1 L water) for 24 h were harvested through three sheets of Miracloth for RT-qPCR and ChIP-qPCR assays.

### Generation of the BP1^ΔIDR1^-C and BP1^ΔIDR2^-C strains

For complementation assays using BP1 lacking IDR1 or IDR2, the deletion variant constructs *BP1*^*ΔIDR1*^ and *BP1*^*ΔIDR2*^ were generated using PH-1 genomic DNA as a template. The fragments encoding BP1^ΔIDR1^ and BP1^ΔIDR2^, together with the native *BP1* promoter, were amplified by PCR with primers listed in Additional file 2: Table S5. The amplicons were then cloned into XhoI-digested pYF11-gfp-G418 (G418; geneticin selectable marker) plasmid. The resulting *BP1*^*ΔIDR1*^*-GFP* and *BP1*^*ΔIDR2*^*-GFP* constructs carrying the G418 resistance marker were individually transformed into the Δ*BP1* mutant using a polyethylene glycol (PEG)-mediated protoplast transformation method. G418-resistant transformants BP1^ΔIDR1^-C and BP1^ΔIDR2^-C strains were identified by PCR and immunoblot assays.

### Confocal microscopy

To observe the subcellular localization of BP1^ΔIDR1^-GFP and BP1^ΔIDR2^-GFP, the BP1^ΔIDR1^-C and BP1^ΔIDR2^-C strains grown on YEPD medium at 25 °C for 24 h were stained with a droplet of 10 μg mL^–1^ DAPI (4′,6-diamidino-2-phenylindole) and imaged with an inverted Zeiss LSM980 confocal laser-scanning microscope (Gottingen, Niedersachsen, Germany). The PH-1 and Δ*BP1* strains accumulating GFP-tagged FgTri1 (encoded by the FGSG_00071 locus) were used as fluorescent reporter strains to observe toxisome formation.

### Protein production and purification

Recombinant plasmids carrying the coding sequence of *BP1* or its variants were constructed and proteins were purified according to previous reports, with modifications [10]. The pET-22a-His-GFP vector harboring *BP1* was transformed into *E. coli* BL21 (DE3) cells. Cells were grown at 37 °C in LB medium containing 50 μg/mL ampicillin to an optical density at 600 nm (OD_600_) of 0.6. Protein production was then induced with β-D-1-thiogalactopyranoside (IPTG) at a final concentration of 0.2 mM, overnight at 16 °C. Cells were harvested by centrifugation for 10 min at 4 °C and then resuspended in lysis buffer (200 mM NaCl, 20 mM MES pH 6.5, and 5% [v/v] glycerol). After sonication, cell debris were removed by centrifugation at 12,000* g* for 60 min. The supernatant was loaded on a His-Trap FF column (GE Healthcare) for fast protein liquid chromatography (FPLC) purification. The proteins were eluted with lysis buffer containing 500 mM imidazole, and fractions with absorbance peaks at OD_280_ were collected. Selected fractions were further purified by cation exchange chromatography (Heparin, HP, GE Healthcare). The proteins were eluted with a linear sodium chloride gradient of up to 1.0 M NaCl. Fractions with absorbance peaks were subjected to SDS-PAGE analysis. Target proteins were subjected to size exclusion chromatography (SEC), and the eluted fraction whose peak corresponded to the protein monomeric state was collected and concentrated using 30-kD molecular mass centrifugal filter devices (Millipore). The MBP trap FF column (GE, Healthcare) was eluted with lysis buffer containing 20 mM maltose. The His-IDR1-GFP, His-IDR2-GFP, His-MGG_09903-GFP, His-NCU07505-GFP, His-BP1, His-Suz12, MBP-BP1, MBP-BP1^ΔIDR1^, and MBP-BP1^ΔIDR2^ proteins were purified using the same protocol as for His-BP1-GFP. Protein concentration was determined using a NanoDrop 2000 (Thermo Scientific), and samples were stored at − 80 °C.

### In vitro phase separation assay

Recombinant fusion proteins were diluted with 50 mM Tris–HCl, pH 7.4, to appropriate concentrations. For liquid–liquid phase separation (LLPS) induction, PEG 8000 (10% [w/v] final concentration) was first added to a solution containing NaCl (125 mM final concentration) in a 0.2-mL Eppendorf tube. Then, target proteins were added (5, 10, 20, or 30 μM final concentration). After incubation, turbidity of the purified protein solutions was observed. To visualize phase-separated proteins, the protein solution (5 μL) mixture was placed onto a glass slide with coverslip. Slides were imaged with an inverted Zeiss LSM980 confocal laser-scanning microscope.

### Fluorescence recovery after photobleaching (FRAP)

FRAP experiments were conducted in vivo and in vitro using a Zeiss LSM 980 confocal laser-scanning microscope. Images were acquired with ZEN software. For FRAP analysis in vivo, the Δ*BP1*-C strain stably expressing *BP1-GFP* was grown on YEPD medium at 25 °C for 24 h before examination. Mycelia were mounted onto glass slides and sealed with coverslips. A region of interest was identified and bleached using a 488-nm laser pulse with 50% power and 2-s dwell time. For FRAP analysis in vitro, the same protocol as for BP1-GFP was used. Briefly, to bleach a region of His-BP1-GFP protein droplets, the laser was set to 100% intensity with a 2-s dwell time. Then, single-section images were captured every 2 s after bleaching. Analysis of the fluorescence intensity of the bleached regions was conducted using the FRAP module in the ZEN image analysis software.

### Time-lapse microscopy

The Δ*BP1*-C strain showing BP1-GFP signal was imaged with a 60 × objective lens. The focus was adjusted, and exposure time and gain were determined based on protein abundance. The region of interest was selected using the built-in software, and images were captured every 1 s. During all measurements, laser power, gain, and field of view were kept constant.

### Yeast two-hybrid assay

For yeast two-hybrid assays, the full-length coding sequence or deletion variants of *BP1* was amplified from first-strand cDNA prepared from strain PH-1 with the corresponding primers (Additional file 2: Table S5). *BP1* sequences were cloned into pGBKT7 at the *Eco*RI restriction site to generate the bait constructs (*BD-BP1*, *BD-BP1*^*ΔIDR1*^, and *BD-BP1*^*ΔIDR2*^). The full-length coding sequence of *Suz12* was inserted into the GAL4 activation domain vector pGADT7 at the *Eco*RI site to generate the prey construct (*AD-Suz12*). The bait and prey plasmids were co-transformed into reporter yeast (*Saccharomyces cerevisiae*) strain AH109 competent cells following the LiAc/ssDNA/PEG (lithium acetate/single-stranded DNA/polyethylene glycol) transformation protocol. After transformants were isolated and grown on synthetic defined (SD) medium without leucine and tryptophan at 30 °C for 3 days, serial dilutions of yeast cells were spotted onto SD medium lacking leucine, tryptophan, histidine, and adenine to assess interactions.

### Co-immunoprecipitation (Co-IP) assays

To construct the *Suz12-Flag* cassette, the full-length *Suz12* coding sequence was amplified with primers listed in Additional file 2: Table S5. The resulting *Suz12-Flag* cassette was verified by DNA sequencing. The *BP1-GFP* and *Suz12-Flag* fusion constructs were then co-transformed into PH-1. Using the same strategy, *F. graminearum* strains co-expressing *BP1*^*ΔIDR1*^-*GFP*/*Suz12-Flag* and *BP1*^*ΔIDR2*^*-GFP/Suz12-Flag* constructs were generated. The resulting strains were examined for GFP and Flag signals by immunoblot analysis with a polyclonal anti-Flag (Sigma, St. Louis, MO) or an anti-GFP antibody (Abcam, Cambridge, UK). For Co-IP assays, total proteins were extracted and incubated with anti-Flag (Abmart, Shanghai, China) agarose beads. Proteins eluted from the Flag agarose were analyzed by immunoblotting with an anti-GFP antibody. Each protein sample was also analyzed with an anti-GAPDH antibody as an internal control.

### Pull-down assay

Recombinant His-Suz12, MBP-BP1, MBP-BP1^ΔIDR1^, and MBP-BP1^ΔIDR2^ were produced in *E. coli* BL21 (DE3) cells. The His-Suz12 supernatant was mixed with 50 μL Ni sepharose 6 Fast Flow beads (GE Healthcare) and incubated at 4 °C for 2 h. The recombinant His-Suz12-bound Ni sepharose beads were incubated with MBP-BP1, MBP-BP1^ΔIDR1^, or MBP-BP1^ΔIDR2^ at 4 °C for another 4 h with gentle shaking. Finally, the beads were washed five times with 1 × phosphate-buffered saline (PBS) buffer. Eluted proteins were then analyzed by immunoblotting with monoclonal anti-His and monoclonal anti-MBP antibodies to detect His-Suz12 and MBP-BP1, MBP-BP1^ΔIDR1^, or MBP-BP1^ΔIDR2^, respectively.

### Peptide pull-down and isothermal titration calorimetry (ITC) assays

For peptide pull-down assays, 62.5 μL of a magnetic streptavidin bead slurry (S1420S, NEB, New England Biolabs) was pre-washed three times with 1 mL peptide binding buffer (50 mM Tris–HCl, pH 8.0, 300 mM NaCl, and 0.1% [v/v] Nonidet P-40). Then, 1.5 μg of biotinylated histone peptides was incubated with streptavidin beads in 1 mL of peptide binding buffer for 1 h at 4 °C. Peptide-bound beads were washed twice with 1 mL of peptide binding buffer and incubated with 1.5 μg of MBP-BP1, MBP-BP1^ΔIDR1^, or MBP-BP1^ΔIDR2^ in 0.5 mL of binding buffer for 3 h at 4 °C. After washing the beads five times with binding buffer, the protein–bead complex was boiled in SDS loading buffer and detected with an anti-MBP antibody. ITC binding experiments were performed as previously described [10].

### ChIP-qPCR

ChIP was performed according to a previously published study, with modifications. Briefly, fresh mycelia of PH-1, BP1^ΔIDR1^-C, and BP1^ΔIDR2^-C strains grown in YEPD medium for 24 h were crosslinked with 1% (w/v) formaldehyde for 20 min. Crosslinking was stopped by adding 125 mM glycine to the sample and incubating for 5 min at 25 °C. Samples were ground in liquid nitrogen and resuspended in lysis buffer (250 mM HEPES pH 7.5, 150 mM NaCl, 1 mM EDTA, 1% [v/v] Triton, 0.1% [w/v] deoxycholate, and 10 mM DTT) containing protease inhibitor (Sangon Co., Shanghai, China). Immunoprecipitation was conducted using anti-H3K27me3 (39,155, Active Motif). Immunoprecipitated proteins were captured by protein A agarose (sc-2001, Santa Cruz, CA, USA) and thoroughly washed to remove non-specific DNA fragments. The precipitated and input DNA fragments were recovered by phenol–chloroform extraction and dissolved in sterile water. The recovered DNA was used as a template for ChIP-qPCR. All primers used for ChIP-qPCR are listed in Additional file 2: Table S5.

### RNA-seq analysis

For RNA-seq sequencing, the wild-type PH-1 and BP1^ΔIDR2^-C strain was incubated in YEPD liquid medium with agitation (180 rpm) for 24 h at 25 °C. The fresh mycelial samples were harvested from liquid medium and ground in liquid nitrogen. Total RNA was isolated from three independent biological replicates from the wild-type PH-1 and BP1^ΔIDR2^-C. DNA-free total RNA was generated by the NEBNext® Ultra™ RNA Library Prep Kit for Illumina® (NEB, USA) following the manufacturer’s recommendations. The library was sequenced on an Illumina Novaseq platform with paired end reads (150 bp) by Novogene Corporation (Beijing, China). The sequencing reads were mapped to the *F. graminearum* PH-1 genome sequence using Hisat2. Feature Counts v1.5.0-p3 was used to count the reads mapped to each gene. Genes with log2 Fold change ≥ 1 or ≤  − 1 and *P*-value ≤ 0.05 were divided into differentially expressed.

### Statistical analysis

Statistical analysis was performed with GraphPad Prism. Data were analyzed by one-way analysis of variance (ANOVA) followed by Fisher’s least significant difference (LSD) test (*P* < 0.05). Different lowercase letters denote significant differences.

### Supplementary Information

**Additional file 1: Fig. S1.** The statistics analysis of BP1 protein phase separation in vitro and in vivo. **Fig. S2.** 1,6-Hexanediol disturbs BP1 protein phase separation in vitro and in vivo. **Fig. S3.** His-BP1 protein phase separation assays. **Fig. S4.** The two IDRs of BP1 undergo phase separation in vitro. **Fig. S5.** Identification of truncated BP1^ΔIDR1^-C and BP1^ΔIDR2^-C strains. **Fig. S6.** H3K27me3 regulates transcriptional repression of DON biosynthesis genes.

**Additional file 2: Table S1.** RNA-seq data of the BP1^ΔIDR2^-C strain. **Table S2.** Overlap between upregulated genes in Δ*Kmt6 *and BP1^ΔIDR2^-C; Δ*BP1* and BP1^ΔIDR2^-C; or Δ*Kmt6*, Δ*BP1*, and BP1^ΔIDR2^-C strains. **Table S3.** Overlap between upregulated genes and H3K27me3-enriched genes among Δ*Kmt6*, Δ*BP1*, and BP1^ΔIDR2^-C strains. **Table S4.** RNA-seq data of *PKS*, *NRPS*, and *P450*-associated genes in Δ*BP1 *and BP1^ΔIDR2^-C strains. **Table S5.** Primers used in this study.

**Additional file 3.** Uncropped images for the blots in Fig. 1H, Fig. 2A, Fig. 5B, Fig. 5D-F, Fig. S3A, Fig. S4A, Fig. S5A, and Fig. S5C.

**Additional file 4.** Review history.

## Data Availability

All data generated or analyzed during this study are included in this published article and its supplementary information files. RNA-seq sequencing data generated during the current study have been deposited at the National Center for Biotechnology Information (NCBI) Sequence Read Archive (SRA) (https://www.ncbi.nlm.nih.gov/bioproject/PRJNA961213) under project accession numbers PRJNA961213 [62]. The published RNA-seq and ChIP-seq data of *F. graminearum* were downloaded from NCBI SRA database under PRJNA663410 [10]. No other scripts and software were used other than those mentioned in the “Methods” section.
