# Supplementary material for: Liquid–liquid phase separation of H3K27me3 reader BP1 regulates transcriptional repression
Source: Genome Biol. 2024 Mar 11;25:67. doi: 10.1186/s13059-024-03209-7 (PMC10926671; doi:10.1186/s13059-024-03209-7)
Supplement: Supplementary file 1 — Additional file 1: Fig. S1. The statistics analysis of BP1 protein phase separation in vitro and in vivo. Fig. S2. 1,6-Hexanediol disturbs BP1 protein phase separation in vitro and in vivo. Fig. S3. His-BP1 protein phase separation assays. Fig. S4. The two IDRs of BP1 undergo phase separation in vitro. Fig. S5. Identification of truncated BP1ΔIDR1-C and BP1ΔIDR2-C strains. Fig. S6. H3K27me3 regulates transcriptional repression of DON biosynthesis genes. [file 13059_2024_3209_MOESM1_ESM.docx]

**Additional file 1: Supplementary figures**

**Title:** **Liquid**–**liquid phase separation of H3K27me3 reader BP1** **regulates transcriptional repression**

**Authors:** Guangfei Tang^1^, Haoxue Xia^1^, Yufei Huang^1,2^, Yuanwen Guo^1^, Yun Chen^3^, Zhonghua Ma^3^, Wende Liu^1^*

**Affiliations:**

^1^ State Key Laboratory for Biology of Plant Diseases and Insect Pests, Institute of Plant Protection, Chinese Academy of Agricultural Sciences, Beijing 100193, China

^2^ College of Plant Protection, Shenyang Agricultural University, Shenyang 110866, China

^3^ State Key Laboratory of Rice Biology, Key Laboratory of Molecular Biology of Crop Pathogens and Insects, Institute of Biotechnology, Zhejiang University, Hangzhou 310058, China

*To whom correspondence may be addressed. Email: [liuwende@caas.cn](mailto:liuwende@caas.cn)

**
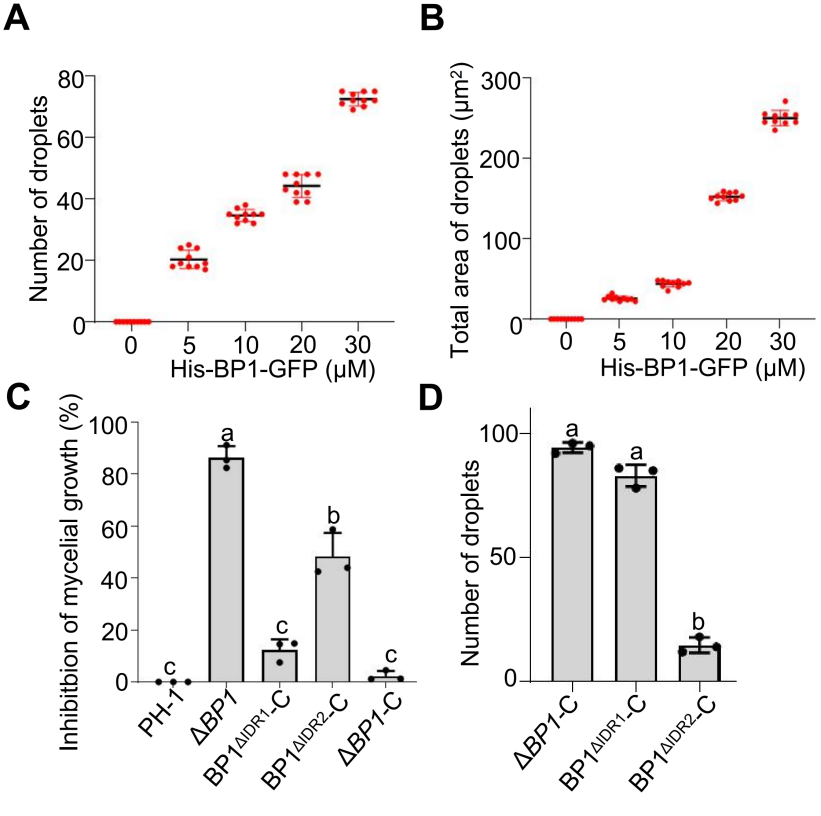
**

**Additional file 1: Fig. S1 The statistics analysis of BP1 protein phase separation *in vitro* and *in vivo*.**

(**A**, **B**) His-BP1-GFP droplet formation is concentration-dependent. Quantification of His-BP1-GFP droplet numbers (**A**) and droplet area (**B**) with increasing His-BP1-GFP concentration (0, 5, 10, 20, and 30 μM). (**C**) Inhibition of mycelial growth for the BP1^ΔIDR1^-C and BP1^ΔIDR2^-C strains shown in (**Fig. 3K**), with levels in PH-1 set to 1. Different lowercase letters denote significant differences at *P* = 0.05. (**D**) Number of fluorescent nuclear puncta from the *ΔBP1*-C, BP1^ΔIDR1^-C, and BP1^ΔIDR2^-C strains shown in (**Fig. 3L**). Different lowercase letters denote significant differences at *P* = 0.05.


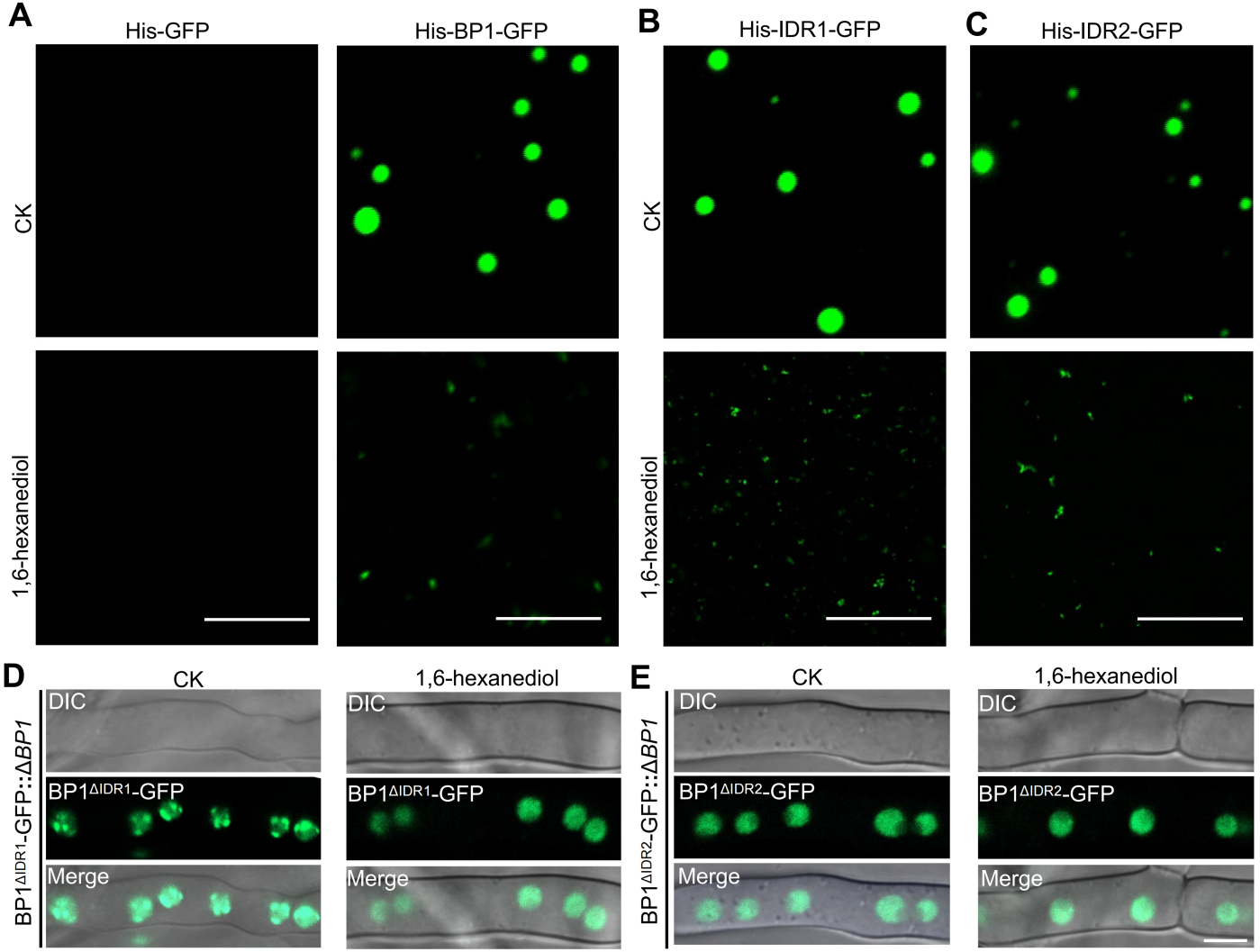


**Additional file 1: Fig. S2 1,6-Hexanediol disturbs BP1 protein phase separation *in vitro* and *in vivo*.**

(**A**–**C**) Droplet formation of recombinant His-BP1-GFP (**A**), His-IDR1-GFP (**B**), and His-IDR2-GFP (**C**) in the presence or absence of 5% (w/v) 1,6-hexanediol *in* *vitro*. Water was used for the untreated control (CK). (**D**, **E**) GFP signals for BP1^ΔIDR1^-GFP (**D**) and BP1^ΔIDR2^-GFP (**E**) nuclear puncta in the Δ*BP1* mutant in the absence or presence of 5% (w/v) 1,6-hexanediol.


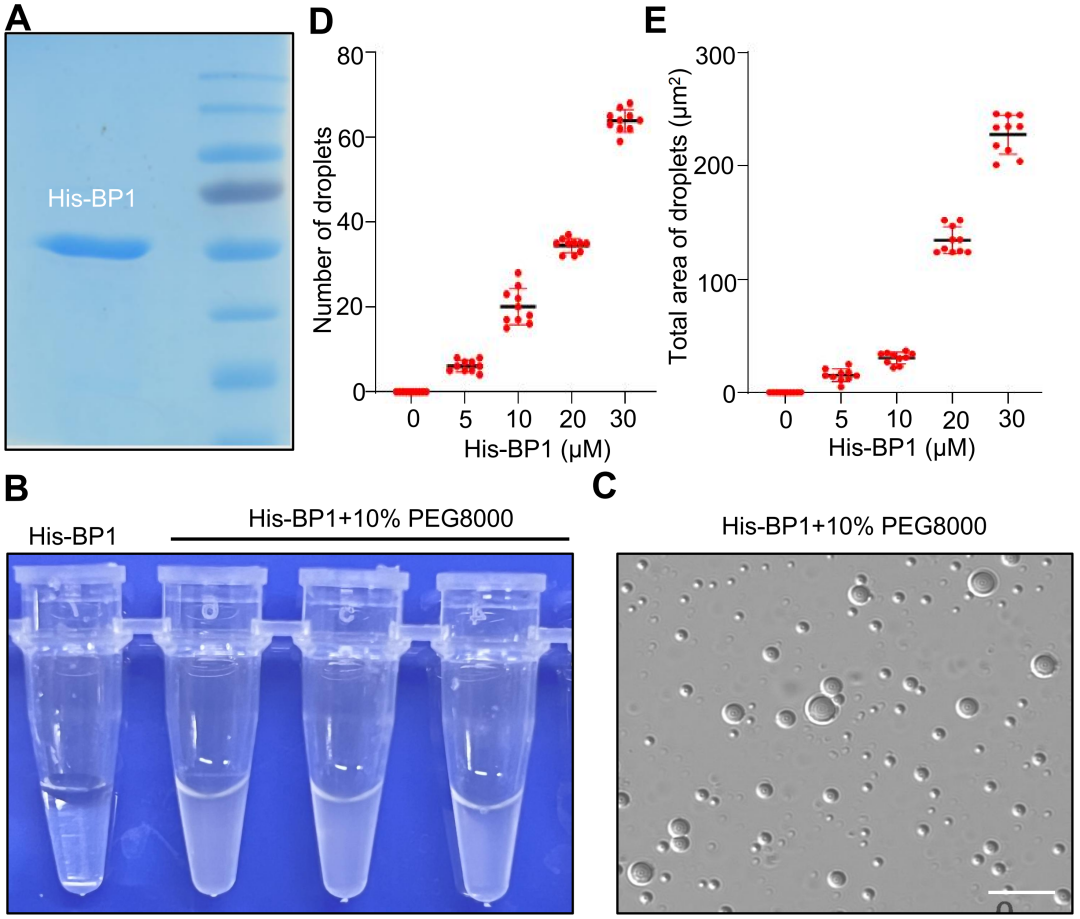


**Additional file 1: Fig. S3 His-BP1 protein phase separation assays**

**A** Coomassie Brilliant Blue staining of recombinant His-BP1 purified from *E. coli*. **B** Turbidity assay of His-BP1 in 10% (w/v) PEG 8000. **C** Representative micrograph of His-BP1 (30 μM) protein preparation. **D**, **E** Quantification of His-BP1 droplet number (**D**) and droplet area (**E**).


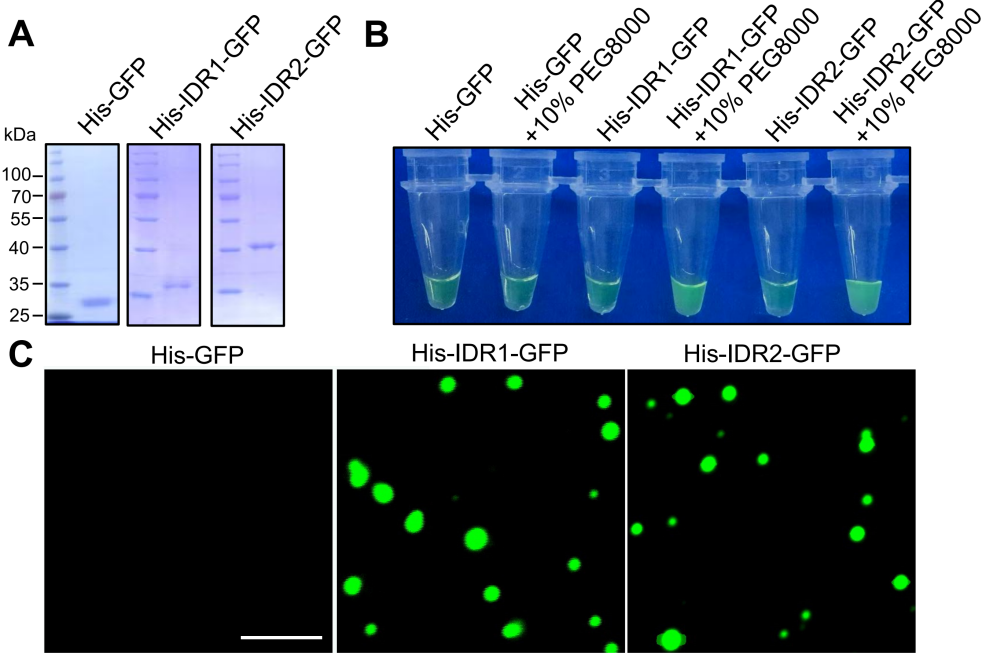


**Additional file 1: Fig. S4** **The two IDRs of BP1 undergo phase separation *in vitro*.**

**A** Coomassie Blue staining of recombinant purified His-GFP, His-IDR1-GFP and His-IDR2-GFP. **B** Turbidity of His-GFP, His-IDR1-GFP, and His-IDR2-GFP droplets in solution in the absence or presence of 10% PEG 8000. **C** Representative fluorescence images of His-IDR1-GFP and His-IDR2-GFP condensates. His-GFP was used as a control.


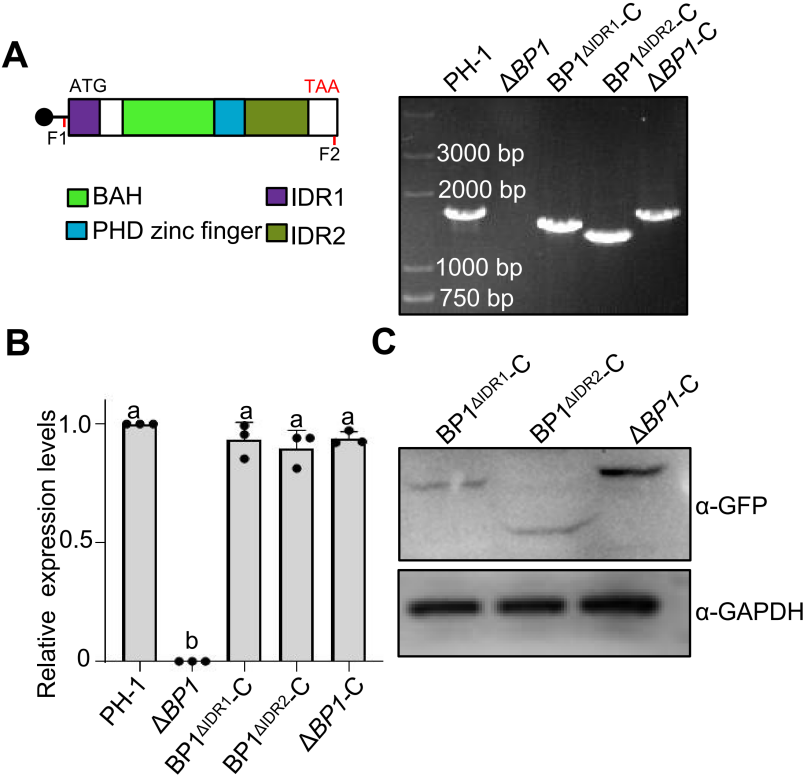


**Additional file 1: Fig. S5 Identification of truncated** **BP1^ΔIDR1^-C and BP1^ΔIDR2^-C strains.**

**A** PCR identification of BP1^ΔIDR1^-C and BP1^ΔIDR2^-C strains. The Δ*BP1* and Δ*BP1*-C strains were used as negative and positive controls, respectively. F1 and F2 shown in the left panel indicate the positions of the primers used for PCR. **B** Relative *BP1* transcript levels in BP1^ΔIDR1^-C and BP1^ΔIDR2^-C strains as determined by RT-qPCR, with PH-1 levels set to 1. Relative transcript levels were normalized to *ACTIN* as the internal standard and presented as means ± SD from three independent experiments. Different lowercase letters denote significant differences at *P* = 0.05 based on one-way ANOVA test. **C** Immunoblot analysis showing BP1^ΔIDR1^-GFP and BP1^ΔIDR2^-GFP abundance in the Δ*BP1* mutant background using an anti-GFP antibody. The full length of BP1-GFP in Δ*BP1*-C strain as the control.

**
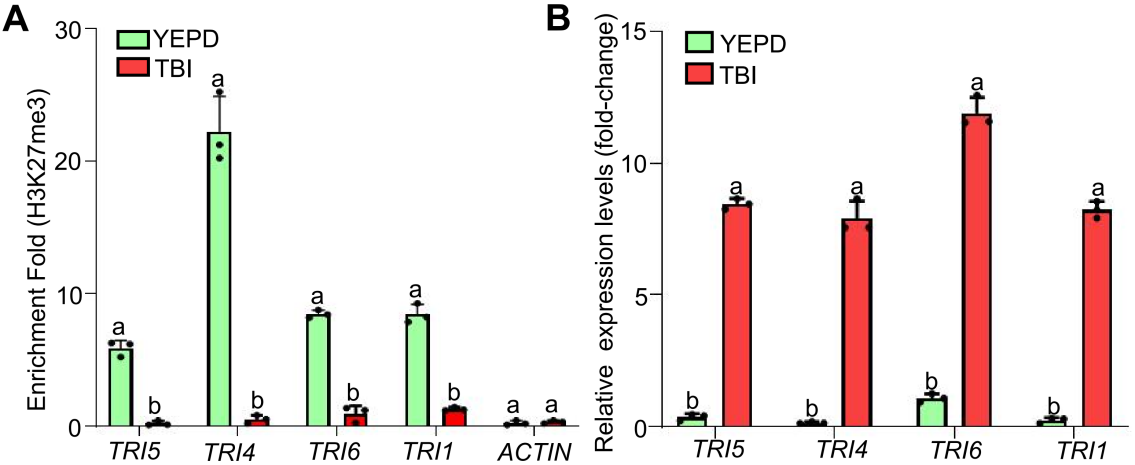
**

**Additional file 1: Fig. S6 H3K27me3 regulates transcriptional repression of DON biosynthesis genes.**

**A** ChIP-qPCR analysis of H3K27me3 enrichment at *TRI* loci (*TRI5*, *TRI4*, *TRI6*, and *TRI1*) after 48 h in toxin-inducing conditions (TBI) or toxin non-inducing conditions (YEPD) using wild-type PH-1 strain. Different lowercase letters denote significant differences at *P* = 0.05. **B** Relative transcript levels of *TRI* (*TRI5*, *TRI4*, *TRI6*, and *TRI1*) genes in YEPD and TBI media as analyzed by RT-qPCR. Transcript levels were normalized to *ACTIN*, with levels in PH-1 set to 1. Different lowercase letters denote significant differences at *P* = 0.05.
