# Supplementary material for: Liquid–liquid phase separation of H3K27me3 reader BP1 regulates transcriptional repression
Source: Genome Biol. 2024 Mar 11;25:67. doi: 10.1186/s13059-024-03209-7 (PMC10926671; doi:10.1186/s13059-024-03209-7)
Supplement: Supplementary file 3 — Additional file 3. Uncropped images for the blots in Fig. 1H, Fig. 2A, Fig. 5B, Fig. 5D-F, Fig. S3A, Fig. S4A, Fig. S5A, and Fig. S5C. [file 13059_2024_3209_MOESM3_ESM.docx]

Additional file 3: Uncropped images for the blots in Fig. 1H, Fig. 2A, Fig. 5B, Fig. 5D-F, Fig. S3A, Fig. S4A, Fig. S5A, and Fig. S5C

Uncropped western blotting and gel for Fig. 1H


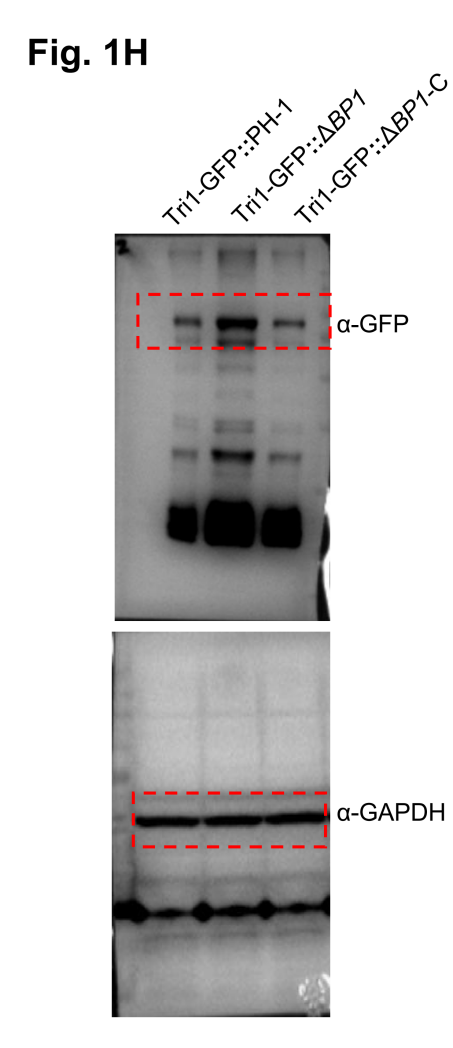


Uncropped image for Fig. 2A


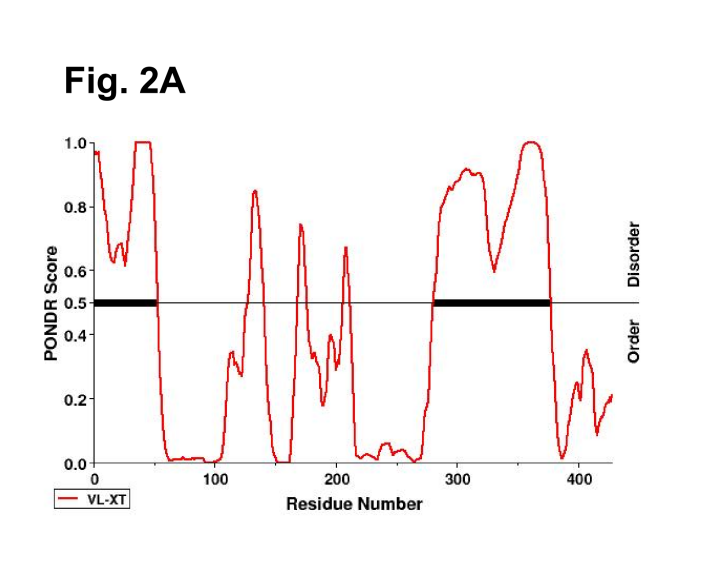


Uncropped western blotting and gel for Fig. 5B,D-F


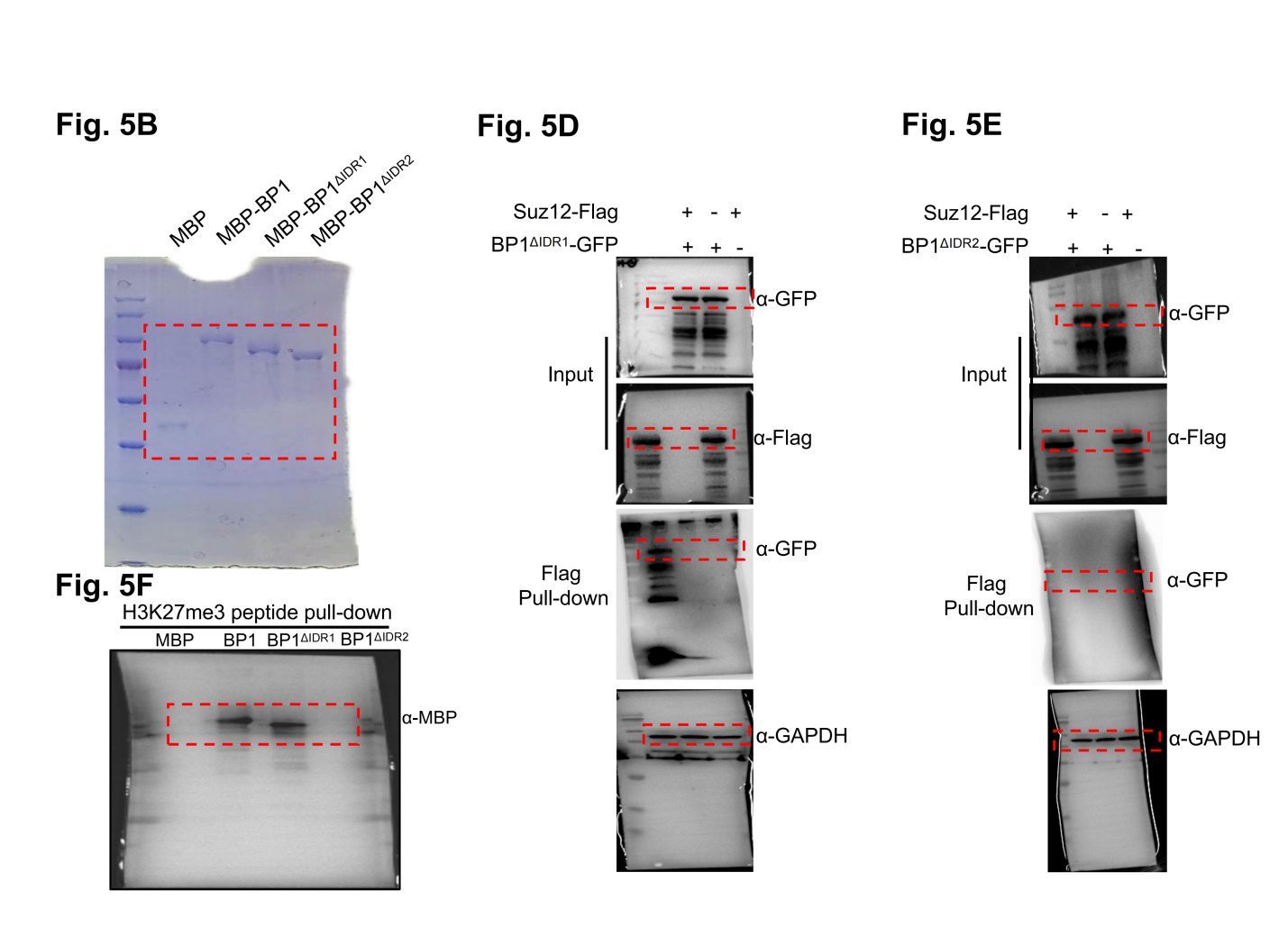


Uncropped gel for Fig. S3A


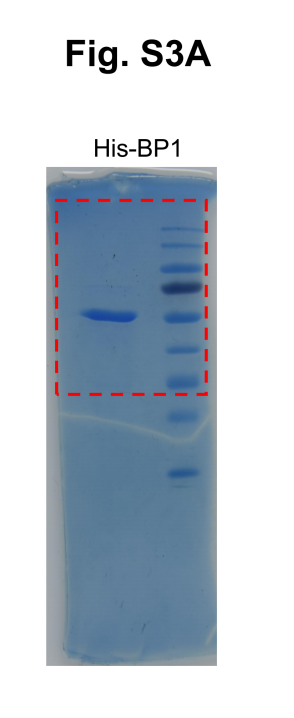


Uncropped gel for Fig. S4A


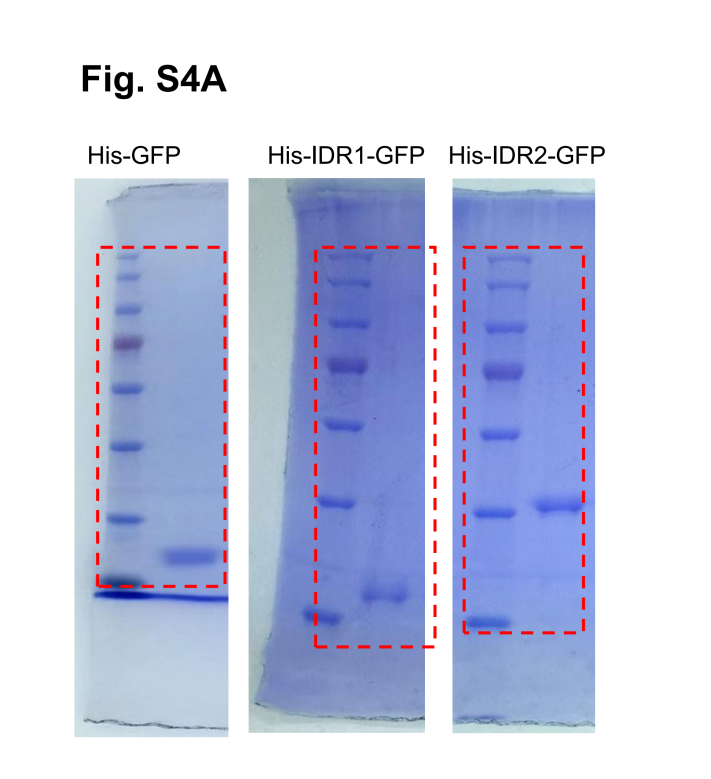


Uncropped western blotting and gel for Fig. S5A, S5C

**
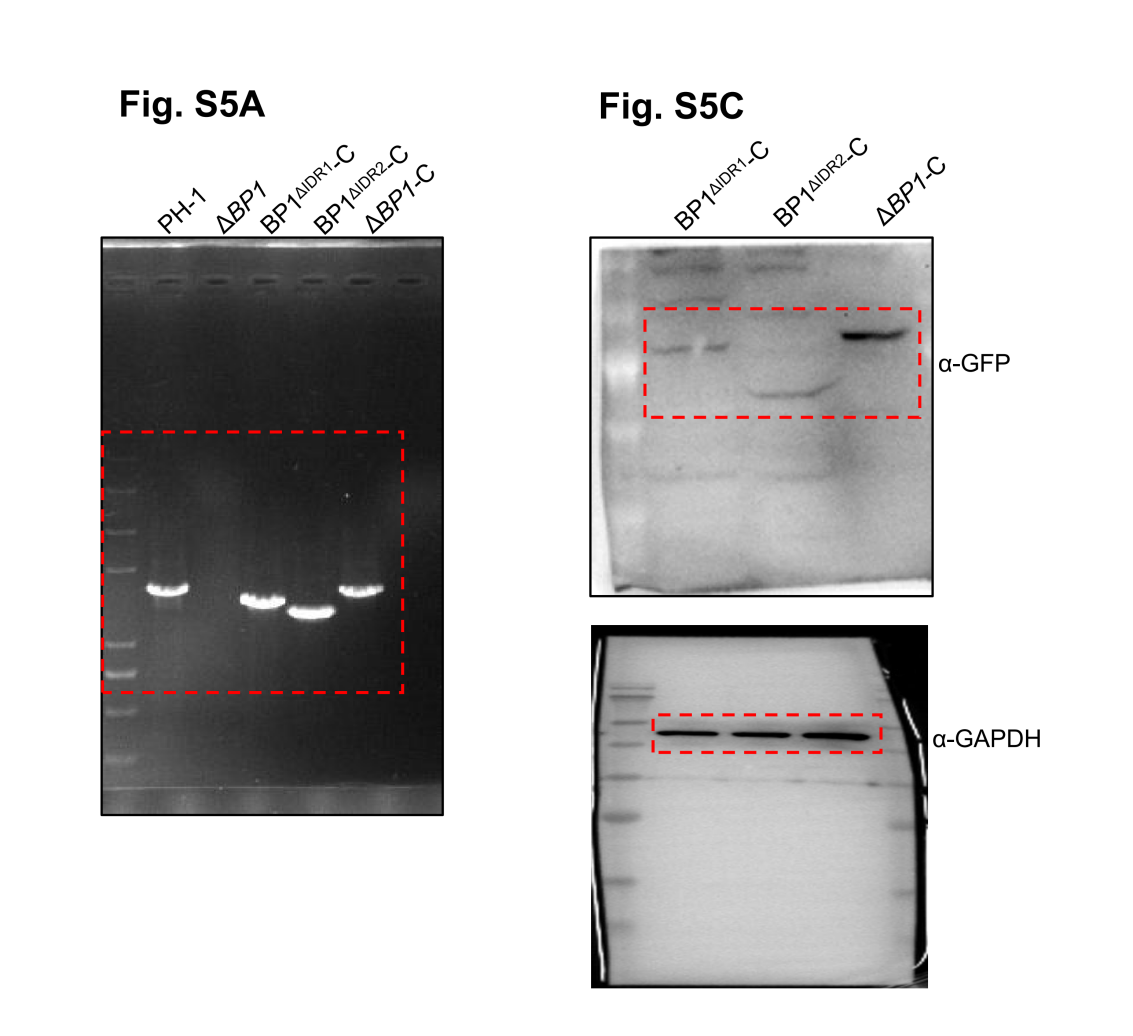
**
