# Supplementary material for: Liquid–liquid phase separation of H3K27me3 reader BP1 regulates transcriptional repression
Source: Genome Biol. 2024 Mar 11;25:67. doi: 10.1186/s13059-024-03209-7 (PMC10926671; doi:10.1186/s13059-024-03209-7)
Supplement: Supplementary file 4 — Additional file 4. Review history. [file 13059_2024_3209_MOESM4_ESM.doc]

Review History

**First round of review**

**Reviewer 1**

**Were you able to assess all statistics in the manuscript, including the appropriateness of statistical tests used?**

There are no statistics in the manuscript.

**Comments to author:**

The protein known as Bromo-adjacent homology (BAH)-plant homeodomain (PHD) domain containing protein 1 (BP1) plays a crucial role in recognizing H3K27 methylation within the cereal fungal pathogen Fusarium graminearum. Previous research has demonstrated that BP1 interacts with the core component of PRC2, Suz12, and directly binds to methylated H3K27. BP1 is primarily found in genomic regions marked by H3K27me3, where it collaborates to suppress gene transcription. In this study, the authors reveal a new aspect of BP1's function, demonstrating that it undergoes phase separation to form liquid condensates, a process essential for PRC2-mediated gene silencing. While these findings are intriguing, it is important to note that some critical control experiments are missing in certain parts of the research and should be addressed. In all the figures, data for WT, BP1 knockout, BP1 IDR1 and IDR2 mutants should be included but they are often missing,

1) Figure1G - Tri-GFP expression in BP-1C strain is missing, could this be included?

2) Figure 2F, 2G and 2H, data for BP1-IDR1 and IDR2 mutants should be included - to inform impact of IDR1/2 mutations on growth and recovery form FRAP.

3) Growth inhibition assays in Fig 2G and Fig S1C - they appeared very different, and the scales on Y axis are different. This is confusing.

4) Fig S1 and S3 - could data for number of droplets formed and total areas of droplets for BP1 KO, BP1-C, BP1-IDR1 and IDR2 mutants be included here for comparison?

5) In Figure 3E and 3F, the information pertaining to droplet formation in BP1-IDR1 and IDR2 mutants was not presented alongside the data for BP1 His-GFP in a comparative manner. It is recommended that these datasets be included side by side within Figure 3 for clarity.

Additionally, the data for FRAP (depicted in Figure 3G-I) has not been displayed for the BP1-IDR1 and IDR2 mutants. These datasets should also be incorporated into the figure for a comprehensive analysis.

6) The same with figures S2 and S4, as data for droplet formation for BP1-IDR1 and IDR2 mutants were also not included?

7) In Figure 4, SUZ12 Flag failed to interact with BP1 IDR1 GFP, but why the growth of BP1 IDR1 GFP not affected (Figure 3K)? Also, how does these binding assays reconcile with previous findings that BAH domain is required for the recruitment of SUZ12?

8) Little or no information on statistical tests? It is unclear how they were calculated.

**Reviewer 2**

**Were you able to assess all statistics in the manuscript, including the appropriateness of statistical tests used?**

Yes, and I have assessed the statistics in my report.

**Comments to author:**

Tang et al. describe BP1, a reader protein of H3K27me3, can form condensates in vitro and in vivo. They identify that the IDR2 of BP is responsible for this clustering. They further find out that the IDR2 deletion is important for transcription repression and DON mycotoxin synthesis. The topic is interesting; however, the current data is not sufficient to support their claims. Particularly, in principle, any proteins can form condensates in vitro. The authors should provide compelling evidence that BP1 forms condensates through LLPS.

Points

1. In the Introduction section, why do the authors think transcription is at "a much faster and with less energy"?

2. The data show that a fraction of proteins within condensates is dynamic whereas the other portion (about 50%) remains un-exchangeable. The authors should rephrase their conclusion.

3. The authors should provide evidence that the target genes are localized within condensates.

4. BP1 is the reader for H3K27me3 whereas H1 is a general repressor. Why does H3K27me3 completely colocalizes with H1?

5. BP1 clusters in cells. There are many potential mechanisms that can lead to the clustering. Additional evidence should be provided if the authors believe the BP1 clustering is caused by phase separation.

6. Endogenous epigenetic regulatory proteins typically have concentrations below micromolar. Since BP1 forms condensates above micromolar, it remains obscure whether the BP1 clustering is formed by phase separation.

7. Figure 1B, I would recommend that the authors add gene names next the bar graph.

8. Figure 1B-E, could the authors explain why they did not include TRI101 in the figures?

9. Figure 2B-D, BP1-GFP shows irregular distributions. There are no liquid-droplet-like structure within cells. These data does not support BP1 form condensates through phase separation.

10. Figure 2J, the authors should quantify their FRAP results and indicate how many cells they have bleached.

11. Figure 3E-F, does BP1 form condensates without PEG? The authors should quantify the saturation concentration of BP1? How is the salt-dependence of BP1 condensate formation?

12. Figure 4, the authors have shown that BP1 interacts with SUZ13 in vitro and in overexpression systems. The authors should provide evidence that endogenous BP1 interacts endogenous SUZ12. Depletion of IDR2 should disrupt this interaction endogenously.

**Authors Response**

**Point-by-point responses to the reviewers’ comments:**

Reviewer reports:
Reviewer #1: The protein known as Bromo-adjacent homology (BAH)-plant homeodomain (PHD) domain containing protein 1 (BP1) plays a crucial role in recognizing H3K27 methylation within the cereal fungal pathogen *Fusarium graminearum*. Previous research has demonstrated that BP1 interacts with the core component of PRC2, Suz12, and directly binds to methylated H3K27. BP1 is primarily found in genomic regions marked by H3K27me3, where it collaborates to suppress gene transcription. In this study, the authors reveal a new aspect of BP1's function, demonstrating that it undergoes phase separation to form liquid condensates, a process essential for PRC2-mediated gene silencing. While these findings are intriguing, it is important to note that some critical control experiments are missing in certain parts of the research and should be addressed. In all the figures, data for WT, BP1 knockout, BP1 IDR1 and IDR2 mutants should be included but they are often missing,

1) Figure1G - Tri1-GFP expression in ΔBP1-C strain is missing, could this be included?

***Response****: We thank the reviewer for these valuable suggestions.* *As suggested, we tested Tri1-GFP accumulation in the* *ΔBP1-C strain. As shown in Fig. 1F of the revised manuscript, the complementation strain ΔBP1-C and wild-type PH-1 formed very faint toxisomes compared to ΔBP1. We quantified the intensities of Tri1-GFP signals in each strain using ImageJ software* *(Fig. 1G). In addition, we measured the abundance of Tri1-GFP in the above strains by immunoblotting (Fig. 1H). We conclude that Tri1-GFP is only highly abundant in the ΔBP1 strain.*

2) Figure 2F, 2G and 2H, data for BP1-IDR1 and IDR2 mutants should be included - to inform impact of IDR1/2 mutations on growth and recovery form FRAP.

***Response****: We thank the reviewer for the constructive comment. As suggested, to further investigate the effect of IDR1/2 mutations on growth, we introduced the BP1^ΔIDR1^-GFP and BP1^ΔIDR2^-GFP cassettes individually into the ΔBP1 strain, generating the complementation strains BP1^ΔIDR1^-C and BP1^ΔIDR2^-C.* *As shown in Fig. 4D, the BP1^ΔIDR1^-C strain showed a growth phenotype similar to that of wild-type PH-1. The BP1^ΔIDR2^-GFP construct failed to complement the growth defect of the ΔBP1 strain.*

*We also tested these complementation strains via fluorescence recovery after photobleaching (FRAP) to assess the consequences of deleting IDR1 or IDR2. As shown in Fig. 4F (upper panels), BP1^ΔIDR1^-GFP formed many* *nuclear puncta. By contrast, BP1^ΔIDR2^-GFP exhibited a* *diffuse nuclear localization in mycelia, lacking a clear punctate pattern (Fig. 4F lower panels). FRAP experiments revealed quick recovery of* *BP1^ΔIDR1^-GFP* *nuclear puncta after targeted photobleaching. On the contrary, the diffuse nuclear localization of BP1^ΔIDR2^-GFP remained low after targeted photobleaching (Fig. 4G, H).*

3) Growth inhibition assays in Fig 2G and Fig S1C - they appeared very different, and the scales on Y axis are different. This is confusing.

***Response****: Thank you for pointing this out. We revised the scales on the y-axes of Fig. 2G and Fig. S1C, as suggested.*

4) Fig S1 and S3 - could data for number of droplets formed and total areas of droplets for BP1 KO, BP1-C, BP1-IDR1 and IDR2 mutants be included here for comparison?

***Response****: Thank you for this comment. To assess the effects of deleting IDR1 or IDR2 on BP1 phase separation, we generated His-BP1^ΔIDR1^-GFP and His-BP1^ΔIDR2^-GFP constructs and purified* *the corresponding recombinant proteins from E. coli (Fig. 4A). We observed droplet formation of His-BP1^ΔIDR1^-GFP and His-BP1^ΔIDR2^-GFP in vitro. Surprisingly, as shown in Fig. 4B, C, His-BP1^ΔIDR1^-GFP and His-BP1^ΔIDR2^-GFP failed to form droplets, preventing us from counting the number of droplets formed and their total areas.* *These results are consistent with our previous finding that His-IDR1-GFP and His-IDR2-GFP formed many round fluorescent droplets based on microscopy observations* *(Additional file 1: Fig. S4B, C). These results indicate that IDR is important for BP1 phase separation in vitro.*

5) In Figure 3E and 3F, the information pertaining to droplet formation in BP1-IDR1 and IDR2 mutants was not presented alongside the data for BP1 His-GFP in a comparative manner. It is recommended that these datasets be included side by side within Figure 3 for clarity.

Additionally, the data for FRAP (depicted in Figure 3G-I) has not been displayed for the BP1-IDR1 and IDR2 mutants. These datasets should also be incorporated into the figure for a comprehensive analysis.

***Response****:* *Thank you for this comment.* *As mentioned in the response to point #4, the protein separation activity of His-BP1^ΔIDR1^-GFP and His-BP1^ΔIDR2^-GFP was abolished (Fig. 4B, C). Therefore, the lack of fluorescent foci for these BP1-IDR deletion variants made it impossible to conduct FRAP.*

6) The same with figures S2 and S4, as data for droplet formation for BP1-IDR1 and IDR2 mutants were also not included?

***Response****:* *Please see our answer to points #4 and #5 above.*

7) In Figure 4, SUZ12 Flag failed to interact with BP1 IDR1 GFP, but why the growth of BP1 IDR1 GFP not affected (Figure 3K)? Also, how does these binding assays reconcile with previous findings that BAH domain is required for the recruitment of SUZ12?
***Response****:* *Thank you for this comment.* *To determine which IDR in BP1 was responsible for the interaction with SUZ12, we performed a co-immunoprecipitation (Co-IP) assay using the wild-type strain carrying the BP1^ΔIDR1^-GFP and Suz12-Flag or BP1^ΔIDR2^-GFP and Suz12-Flag constructs. As shown in Fig. 5D, BP1 lacking IDR1 still interacted with Suz12. However, BP1 lacking IDR2 was unable to do so (Fig. 5E). This result is consistent with the growth phenotypes of the strains, which supported an important role for IDR2 in BP1 function in the vegetative growth of F. graminearum (Fig. 4D).*

*We previously demonstrated that the BAH domain of BP1 directly binds to H3K27me3 to recruit PRC2 and regulate H3K27me3-mediated gene repression. Many BAH domain–containing proteins were reported to be readers of the H3K27me3 mark, such as the BAH domain of BAHD1 in mammals and the BAH and plant homeodomain (PHD) finger-containing proteins SHORT LIFE (SHL) and EARLY BOLTING IN SHORT DAYS (EBS) in Arabidopsis. Therefore, in our previous article, we reported that the BAH domain is responsible for specifically recognizing H3K27me3 to recruit Suz12 (Tang., et al 2021, Nucleic Acids Research. doi:10.1093/nar/gkab844). In the current study, we further showed that BP1 dynamically forms nuclear puncta to undergo LLPS, which depends on its IDR2 region. Mounting evidence has demonstrated that LLPS of proteins enables the highly efficient and reversible adjustment of cellular events such as chromatin organization, transcriptional regulation, and signal transduction. Our results indicate that the IDR2 region of BP1 stimulates its binding activity to H3K27me3 by interacting with Suz12.*

8) Little or no information on statistical tests? It is unclear how they were calculated.

***Response****:* *Thank you for pointing out this gap in our manuscript. Statistical analysis was performed in GraphPad Prism. Data were analyzed by one-way ANOVA followed by Fisher’s LSD test (P < 0.05). Different lowercase letters in the figures represent significant differences. This information was added to the figure legends.*

Reviewer #2: Tang et al. describe BP1, a reader protein of H3K27me3, can form condensates *in vitro* and *in vivo*. They identify that the IDR2 of BP is responsible for this clustering. They further find out that the IDR2 deletion is important for transcription repression and DON mycotoxin synthesis. The topic is interesting; however, the current data is not sufficient to support their claims. Particularly, in principle, any proteins can form condensates in vitro. The authors should provide compelling evidence that BP1 forms condensates through LLPS.

Points

1. In the Introduction section, why do the authors think transcription is at "a much faster and with less energy"?

***Response****:* *We rephrased this statement in the Introduction.*

2. The data show that a fraction of proteins within condensates is dynamic whereas the other portion (about 50%) remains un-exchangeable. The authors should rephrase their conclusion.

***Response****:* *We rephrased this conclusion; thank you for pointing this out.*

3. The authors should provide evidence that the target genes are localized within condensates.

***Response****:* *Thank you for this suggestion. Based on ChIP-seq of BP1-GFP data, we screened* *for target proteins of BP1 that were predicted to localize to the nucleus and observed their co-localization with BP1. In co-localization experiments, we showed that the protein encoded by the target gene FGSG_04782- fused to mCherry localized within condensates and overlapped with the nuclear puncta of BP1-GFP (Figure R1). Your suggestion is very interesting. Indeed, we would like to explore which target gene is localized within BP1 condensates in the future to better reveal the molecular mechanism of the transcriptional regulation of target genes. We will continue to explore this mechanism in future studies and will not elaborate on this point in the current manuscript.We hope you will agree with this change.*

***Figure R1****: Hyphae of strains carrying BP1-GFP and FGSG_04782-mCherry* *constructs examined by epifluorescence microscopy. Scale bar, 5 μm.*

4. BP1 is the reader for H3K27me3 whereas H1 is a general repressor. Why does H3K27me3 completely colocalizes with H1?

***Response****:* *This is a good point. We co-transformed BP1-GFP and H1-mCherry into the wild-type F. graminearum strain only to show that BP1-GFP localizes to the nucleus. We agreed with the reviewer’s point that H1 is a general repressor, whereas BP1 is the reader for H3K27me3, making these results inappropriate or at best confusing. As suggested, we deleted information about the co-localization of BP1-GFP and H1-mCherry in mycelia and protoplasts. Instead, we now show that BP1-GFP localizes to the nucleus based on staining with 4′,6-diamidino-2-phenylindole (DAPI), as shown in Fig. 2B.*

5. BP1 clusters in cells. There are many potential mechanisms that can lead to the clustering. Additional evidence should be provided if the authors believe the BP1 clustering is caused by phase separation.

***Response****: We completely agree with the reviewer’s point that protein clustering can indeed be caused by many factors. To verify the specific molecular mechanism behind BP1 protein clustering, we used the phase separation inhibitor 1,6-hexanediol. Treatment with 1,6-hexanediol significantly inhibited the formation of BP1-GFP puncta in the nucleus (Fig. 2D) and the formation of droplets of recombinant His-BP1-GFP in vitro (Additional file 1: Fig. S2 A). Subsequently, we observed the dynamics of BP1 protein into nuclear puncta and found that BP1* *clustering is indeed dynamic (Fig. 2F). We also confirmed the clustering of BP1-GFP into puncta in the nucleus (Fig. 2G); moreover, recombinant His-BP1-GFP (Fig. 3H) showed recovery of fluorescence after FRAP. Therefore, we suspected that BP1 might undergo protein phase separation. Domain analysis showed that BP1 harbors two IDRs. We conducted phase separation experiments after deleting each IDR individually in vitro, which revealed the loss of phase separation activity after the deletion of IDR1 or IDR2 (Fig. 4B, C). In addition, the deletion of IDR2 seriously affected the clustering of BP1 into puncta in the nucleus (Fig. 4E). FRAP experiments also showed that BP1^ΔIDR1^-GFP in nuclear puncta was dynamically exchanged with BP1^ΔIDR1^-GFP in the surrounding environment after intense laser bleaching (Fig. 4F upper). Furthermore, BP1^ΔIDR1^-GFP in mycelia showed a diffuse nuclear* *localization after 1,6-hexanediol treatment (Additional file 1: Fig. S2D). From these in vivo and in vitro results, we conclude that the clustering of BP1 into nuclear puncta is caused by phase separation.*

6. Endogenous epigenetic regulatory proteins typically have concentrations below micromolar. Since BP1 forms condensates above micromolar, it remains obscure whether the BP1 clustering is formed by phase separation.

***Response****:* *We agree with the reviewer’s statement that endogenous proteins are often present at concentrations below the micromolar range. To confirm that protein clustering is caused by phase separation, we performed FRAP on BP1 puncta (Fig. 2F, G). We also performed treatments with the phase separation inhibitor 1,6-hexanediol, which strongly affected the clustering of BP1 into puncta (Fig. 2D). Finally, we deleted the IDR of BP1 and confirmed in vivo and in vitro that IDR2 is essential for the clustering of BP1 puncta in the nucleus (Fig. 4). Thus, our results demonstrate that BP1 undergoes LLPS to form liquid droplets in vivo and in vitro.*

7. Figure 1B, I would recommend that the authors add gene names next the bar graph.

***Response****:* *Done.*

8. Figure 1B-E, could the authors explain why they did not include TRI101 in the figures?

***Response****:* *Based on* *RNA-seq and RT-qPCR analyses, we determined that the expression level of* *TRI101 was unchanged in the ΔBP1 strain relative to the wild type* *(Figure R2A). Additionally, in ChIP-seq and ChIP-qPCR, TRI101 did not show enriched binding by BP1-GFP compared to the negative control gene ACTIN (Figure R2B). Therefore, we did not include TRI101 in Figure 1B-E.*

***Figure R2****.* ***A*** *Relative TRI101 transcript levels in wild-type PH-1 and ΔBP1, as determined by RT-qPCR. Transcript levels were normalized to ACTIN, with levels in PH-1 set to 1.* ***B*** *ChIP-qPCR analysis of relative BP1-GFP levels at the TRI101 locus.*

9. Figure 2B-D, BP1-GFP shows irregular distributions. There are no liquid-droplet-like structure within cells. These data does not support BP1 form condensates through phase separation.
***Response****:* *Thank you for this comment. We rephrased this statement in the revised text (Line 170, Page 6).*

10. Figure 2J, the authors should quantify their FRAP results and indicate how many cells they have bleached.
***Response****:* *We apologize for the insufficient description of our FRAP analysis. Detailed information has now been provided in the revised figure legends.*

11. Figure 3E-F, does BP1 form condensates without PEG? The authors should quantify the saturation concentration of BP1? How is the salt-dependence of BP1 condensate formation?
***Response****:* *As suggested, we performed protein phase separation assays with increasing concentrations of His-BP1-GFP, ranging from 10 μM to 10 mM, in the absence of PEG to quantify the saturation concentration of BP1. However, even when the BP1 concentration reached the millimolar level, we failed to observe BP1 condensate formation* *(Figure R3A). We also tried to change the salt concentration without adding PEG. His-BP1-GFP was soluble and did not form droplets under different salt conditions (Figure R3B). Therefore, BP1 alone does not undergo LLPS under physiological salt conditions unless a crowding agent such as PEG is added. Several previous findings support our results. For example, human MeCP2 (methyl-CpG binding protein 2) protein, a transcriptional repressor that interacts with methylated DNA and H3K27me3 in neurons, undergoes LLPS in droplet formation buffers containing 10% PEG8000 in vitro independently of the cation used in the buffer (Na^+^, K^+^, NH4^+^) (Wang., et al 2020, Cell Research. doi:* *10.1038/s41422-020-0288-7; Zhang., et al 20222, Nucleus. doi: 10.1080/19491034.2021.2024691).*

***Figure R3****.* ***A*** *Phase separation assay of recombinant His-BP1-GFP at different protein concentrations in the absence of PEG 800.* ***B*** *Phase separation assay of 30 μM His-BP1-GFP at different NaCl concentrations without adding PEG.*

12. Figure 4, the authors have shown that BP1 interacts with SUZ12 *in vitro* and in overexpression systems. The authors should provide evidence that endogenous BP1 interacts endogenous SUZ12. Depletion of IDR2 should disrupt this interaction endogenously.

***Response****: Thank you very much for the positive evaluation of our results. We apologize for the misunderstanding due to the unclear description of Fig. 5D, E. In these panels, we show the results of Co-IP assays in endogenous expression systems. With agree with the reviewer that it is important to understand which IDR of BP1 is responsible for interacting with SUZ12 in vivo. To address this question, we constructed the clones Suz12-Flag, BP1^ΔIDR1^-GFP, and BP1^ΔIDR2^-GFP driven by their respective native promoters. We performed a Co-IP assay in strains carrying BP1^ΔIDR1^-GFP or BP1^ΔIDR2^-GFP and the Suz12-Flag constructs in the endogenous expression system. We confirmed the accumulation of each protein in the strain expressing the two fusion constructs by immunoblot analysis; strains expressing a single fusion construct were used as references. For* *the Co-IP assays, we extracted total proteins and incubated them with anti-Flag agarose. As shown in Fig. 5D, E, BP1 lacking IDR1 still interacted with Suz12. However, BP1 lacking IDR2 was unable to interact with Suz12.*

**Second round of review**

**Reviewer 1**

Overall, the authors have improved the manuscript.

As I have mentioned in the first review, some experiments lacked proper controls. It is noted that controls have been incorporated into some figures, but there are still lacks of controls in some figures, such as in Figure 7 and figure R1.

Specific Points:

Figure 7:

Suggested Improvement: Include data on BP1 IDR1 and IDR1-C mutants in Figure 7. This addition will enhance the comprehensiveness of the analysis.

Figure R1:

The colocalization of FGSG-cherry with BP1 in the condensate is not convincing and appears inconclusive. Enrichment does not seem to occur together. Also, what is the target gene FGSG_04782- how was this gene chosen? Also, there should be inclusion of more of both target and non-target genes. There is also a need to incorporate information and analysis to quantify the co-localization of target genes with BP1.

Furthermore, please clarify and provide additional analysis regarding the colocalization of FGSG cherry with BP1, especially within the condensate. Extend this analysis to include BP1 IDR1, IDR2, IDR1-C, and IDR2-C mutants to ensure a thorough examination.

These specific points should contribute to a more comprehensive and robust presentation of the data.

**Reviewer 2**

The authors have addressed most of my comments. However, one of the main points the authors have not fully addressed is whether clustering of BP1 is driven through LLPS since BP1 does not form condensates on its own even above mM concentration, which is enormously higher than endogenous proteins. Clustering of proteins can be due to either as drivers or clients (Cell Rep. 2023 Oct 31;42(10):113136.). I would recommend that the authors weaken their claims on this subject. At least, they should give alternative hypothesis in their discussion.

**Authors Response**

**Point-by-point responses to the reviewers’ comments:**

Reviewer reports:
Reviewer #1: Overall, the authors have improved the manuscript.
As I have mentioned in the first review, some experiments lacked proper controls. It is noted that controls have been incorporated into some figures, but there are still lacks of controls in some figures, such as in Figure 7 and figure R1.
Specific Points:
Figure 7: Suggested Improvement: Include data on BP1 IDR1 and IDR1-C mutants in Figure 7. This addition will enhance the comprehensiveness of the analysis.
***Response:*** *Thank you for the constructive suggestions. We added the effects of deleting IDR1 on BP1 DON production and virulence in the revised Fig. 7, as suggested.*

Figure R1: The colocalization of FGSG-cherry with BP1 in the condensate is not convincing and appears inconclusive. Enrichment does not seem to occur together. Also, what is the target gene FGSG_04782- how was this gene chosen? Also, there should be inclusion of more of both target and non-target genes. There is also a need to incorporate information and analysis to quantify the co-localization of target genes with BP1.

***Response：****We appreciate the reviewer being thorough in evaluating our manuscript. Following the reviewer’s suggestion that we should be inclusion of more of both target and non-target genes. We analyzed co-upregulated genes in RNA-seq data (ΔKmt6, ΔBP1, and BP1^ΔIDR2^-C) and H3K27me3 enrichment genes, identified a list of total of 1,323 putative target genes (Fig. 6B, C). We selected three genes (Fg00081, Fg08182, Fg04782) from these 1,323 putative target genes that were predicted to localize to the nucleus, and two genes including Actin (Fg07335), and the actin capping protein CapA (Fg08621) that were not in the list as putative non-target genes. First, we verified H3K27me3 enrichment of selected five* *genes by ChIP-qPCR and confirmed they are target and non-target genes (Fig. A below). Then, selected target and non-target genes was fused to mCherry tag and drove the encoding construct by their native promoter, respectively. We transformed this construct and BP1-GFP into F. graminearum, observed their co-localization with BP1. In co-localization experiments, we observed that the protein encoded by the target gene Fg00081, Fg08182, and Fg04782- fused to mCherry localized within condensates and overlapped with the nuclear puncta of BP1-GFP (Fig. B below). Correspondingly, the non-target gene was not localized into the BP1 condensate (Fig. C below). These results further indicate that the condensate of BP1 is important for H3K27me3-targeted gene transcription. Because we focused on the role of BP1 in transcriptional repression in this work, these data will not be presented in the current manuscript. We hope you will agree with us.*

***Figure:*** ***A*** *ChIP-qPCR analysis of H3K27me3 enrichment at selected putative target and non-target gene loci using wild-type PH-1 strain.* ***B, C*** *Hyphae of strains carrying BP1-GFP and target gene (****B****) and non-target gene (****C****) containing mCherry examined by microscopy. The localization of target gene to nuclear puncta is indicated by yellow arrows, Scale bar, 5 μm.*

Furthermore, please clarify and provide additional analysis regarding the colocalization of FGSG cherry with BP1, especially within the condensate. Extend this analysis to include BP1 IDR1, IDR2, IDR1-C, and IDR2-C mutants to ensure a thorough examination.
These specific points should contribute to a more comprehensive and robust presentation of the data.
***Response：****As suggested, we furtherly observed the colocalization of Fg04782-mCherry with BP1^ΔIDR1^-GFP, BP1^ΔIDR2^-GFP. As shown in the figure below, BP1^ΔIDR2^-GFP exhibited a diffuse nuclear localization in mycelia, lacking an obvious colocalization punctate pattern with Fg04782-mCherry. By contrast, BP1^ΔIDR1^-GFP formed many nuclear puncta with Fg04782-mCherry within the condensate. These results further demonstrate that IDR2 is a key region driving the condensate of BP1 in the nucleus. As we responding to your previous question, we think exploring the colocalization of target genes within BP1 condensates is important to reveal the molecular mechanism of the global transcriptional regulation of target* *genes, but we think it is beyond the scope of this manuscript. Therefore, we would like to not include this data in the present study. We sincerely hope that you will agree with us*.

***Figure:*** *The colocalization observation of BP1-GFP, BP1^ΔIDR1^-GFP, BP1^ΔIDR2^-GFP, and Fg04782-mCherry as examined by epifluorescence microscopy in F. graminearum. The localization of Fg04782 to nuclear puncta is indicated by yellow arrows, Scale bar, 5 μm.*

Reviewer #2: The authors have addressed most of my comments. However, one of the main points the authors have not fully addressed is whether clustering of BP1 is driven through LLPS since BP1 does not form condensates on its own even above mM concentration, which is enormously higher than endogenous proteins. Clustering of proteins can be due to either as drivers or clients (Cell Rep. 2023 Oct 31;42(10):113136.). I would recommend that the authors weaken their claims on this subject. At least, they should give alternative hypothesis in their discussion.
*Response: Thank you for pointing this out. We agree with the reviewer’s comments that clustering of BP1 protein may be due to either as drivers or clients. The current data may not fully support the conclusion that clustering of BP1 is driven through LLPS. Following your guidance, we gave alternative hypothesis in the revised discussion (Line 450-456). At the same time, the article (Cell Rep. 2023 Oct 31;42(10):113136.) you mentioned was quoted.*

**Third round of review**

**Reviewer 1**

The authors have addressed all my comments.

**Reviewer 2**

The authors have addressed my concerns. I recommend the acceptance of this manuscript.
